# Appropriate management of acute gastroenteritis in Australian children: A population-based study

**DOI:** 10.1371/journal.pone.0224681

**Published:** 2019-11-07

**Authors:** Neroli Sunderland, Johanna Westbrook, Rachel Urwin, Zoe Knights, Jonny Taitz, Helena Williams, Louise K. Wiles, Charlotte Molloy, Peter Hibbert, Hsuen P. Ting, Kate Churruca, Gaston Arnolda, Jeffrey Braithwaite, on behalf of the CareTrack Kids investigative team

**Affiliations:** 1 Centre for Health Systems and Safety Research, Australian Institute of Health Innovation, Macquarie University, Sydney, NSW, Australia; 2 Emergency Department, The Children’s Hospital at Westmead, Westmead, NSW, Australia; 3 Clinical Excellence Commission, McKell Building, Sydney, NSW, Australia; 4 Australian Commission on Safety and Quality in Health Care, Women’s and Children’s Hospital, SALHN, Adelaide, SA, Australia; 5 Australian Centre for Precision Health, University of South Australia Cancer Research Institute (UniSA CRI), School of Health Sciences, University of South Australia, Adelaide, SA, Australia; 6 South Australian Health and Medical Research Institute (SAHMRI), Adelaide, SA, Australia; 7 Centre for Healthcare Resilience and Implementation Science, Australian Institute of Health Innovation, Macquarie University, Sydney, NSW, Australia; Universitat Witten/Herdecke, GERMANY

## Abstract

**Objectives:**

To determine the proportion of care provided to children with acute gastroenteritis (AGE) in Australia consistent with clinical practice guidelines.

**Methods:**

Indicators were developed from national and international clinical practice guideline (CPG) recommendations and validated by an expert panel. Medical records from children ≤15 years presenting with AGE in three healthcare settings–Emergency Department (ED), hospital admissions and General Practitioner (GP) consultations–from randomly selected health districts across three Australian States were reviewed. Records were audited against 35 indicators by trained paediatric nurses, to determine adherence to CPGs during diagnosis, treatment, and ongoing management.

**Results:**

A total of 14,434 indicator assessments were performed from 854 healthcare visits for AGE by 669 children, across 75 GPs, 34 EDs and 26 hospital inpatient services. Documented adherence to guidelines across all healthcare settings was 45.5% for indicators relating to diagnosis (95% CI: 40.7–50.4), 96.1% for treatment (95% CI: 94.8–97.1) and 57.6% for ongoing management (95% CI: 51.3–63.7). Adherence varied by healthcare setting, with adherence in GPs (54.6%; 95% CI: 51.1–58.1) lower than for either ED settings (84.7%; 95% CI: 82.4–86.9) or for inpatients (84.3%; 95% CI: 80.0–87.9); p<0.0001 for both differences. The difference between settings was driven by differences in the diagnosis and ongoing management phases of care.

**Conclusions:**

Adherence to clinical guidelines for children presenting to healthcare providers with AGE varies according to phase of care and healthcare setting. Although appropriate diagnostic assessment and ongoing management phase procedures are not well documented in medical records (particularly in the GP setting), in the treatment phase children are treated in accordance with guidelines over 90% of the time.

## Introduction

Acute gastroenteritis (AGE) is a common condition that imposes a significant burden on communities and healthcare systems worldwide. In Australia there are an estimated 17.7 million cases of AGE per year, causing 2.7 million visits to health professionals and at a conservatively estimated annual cost to the public health system of AUD$359 million (2016 prices; USD$258 million).[1] Additional costs including private health care charges and over-the-counter medications are borne by individuals, and an estimated 13.1 million days of productivity are lost due to AGE each year in Australia.[2] A large proportion of AGE cases occur in children, with patients <5 years accounting for 13.9% of cases, at a rate of 1.6 cases per person per year,[1] and who require around 250,000 general practitioner consultations.[1] Although the introduction of an effective rotavirus vaccine to the national immunisation program in 2007 has significantly reduced Emergency Department (ED) presentations[3] and hospitalisation rates,[4] AGE remains a leading cause of hospitalisation of children. In Australia in 2007–2010, there were approximately 5,000 hospital admissions for AGE per 100,000 children <5 years of age per year,[4] and a further 230 admissions/100,000 5–19 year olds annually.[4] AGE is responsible for an average of 58 emergency department presentations per 100,000 children <5 years per week in the state of New South Wales alone.[3]

Given the high frequency of both AGE cases and AGE-related healthcare interactions for children, it is imperative that the care received is appropriate and effective. Many commonly used therapeutic interventions for AGE lack evidence of effectiveness, or their benefits are outweighed by the risk of serious side effects. For example, intravenous rehydration is no more effective than oral rehydration for children with mild to moderate dehydration, but is associated with more adverse events, longer hospital stays and increased costs.[5] The anti-diarrheal medication loperamide is not recommended for use in children, despite its effectiveness at reducing the duration of diarrhea by a mean of 0.8 days, due to the risk of side effects such as ileus, abdominal distension, lethargy and sleepiness.[6] Clinical practice guidelines (CPGs) have been developed by various bodies, including the European Society for Paediatric Gastroenterology, Hepatology, and Nutrition (ESPGHAN)[7] and the UK’s National Institute for Health and Clinical Excellence (NICE),[8] to assist health professionals to provide appropriate, evidence-based care to children with AGE. In general, these guidelines promote the use of enteric (oral or nasogastric) rehydration and normal diets, and discourage laboratory tests and medications. There is good evidence that when followed they are clinically effective,[9] and can reduce hospitalisation rates[10] and the costs of care by up to 50%.[11, 12] However, there is significant variation in guideline adherence in practice,[12, 13] and there is limited published literature about how childhood AGE is being treated in Australian hospitals and General Practices (GPs).

The CareTrack Kids (CTK) study assessed care of Australian children aged 0–15 years, in 2012 and 2013, to determine the proportion that received care in line with CPGs for 17 common conditions.[14] We present and discuss the CTK results for AGE, outlining, the proportion of care delivered in accordance with CPGs to children with this condition in Australia, by phase of care and healthcare setting.

## Methods

The CTK methods have been described in detail elsewhere.[14–16] We describe some aspects specifically relevant to AGE, with additional details provided in S1 Appendix.

### Development of indicators

The RAND-UCLA method was modified and applied to develop indicators.[17] A systematic search[16] for national and international CPGs identified eleven CPGs for AGE, from which all recommendations (n = 31) (S2 and S3 Appendices) were extracted for consideration. Recommendations were screened and excluded if they were out of scope (such as structure-level measures), if there was a low likelihood of information being documented in the medical record, or if they did not provide a specific or conclusive action against which compliance could be assessed (e.g. they were guiding statements only with no recommended actions; if they used auxiliary verbs such as “may”, “consider” and “could”).[18] After deleting five such recommendations, 26 were drafted as candidate indicators (commencing with the eligibility criteria followed by the compliance action) and passed to review by clinicians.[18]

Candidate indicators were subjected to three-rounds of internal and external clinical review. Internal review was performed by four clinicians (three paediatricians and one general practitioner) involved in CTK. External review was by three paediatricians external to the project; reviewers were recruited via advertisements and communications with relevant medical colleges and associations. All reviewers recorded whether each candidate indicator was acceptable and feasible to collect, and its level of clinical impact.[16, 18] External reviewers, in addition, used a nine-point Likert scale to score each candidate indicator as representative of appropriate care delivered to children during 2012 and 2013.[17]

During review, candidate indicators were excluded due to low acceptability, feasibility, or impact; if the concept was covered in other indicator(s); or rated with a low appropriateness score by external reviewers. Fourteen of the 26 candidate indicators were removed during internal review, with all 12 passed to external review retained (see S2 Appendix for more detail). These 12 candidate indicators were re-formatted into 35 medical record audit indicator questions (see S3 Appendix for indicator development); all final indicators are summarised in S1 Table.

### Sample size, sampling process and data collection

**Fig 1:**
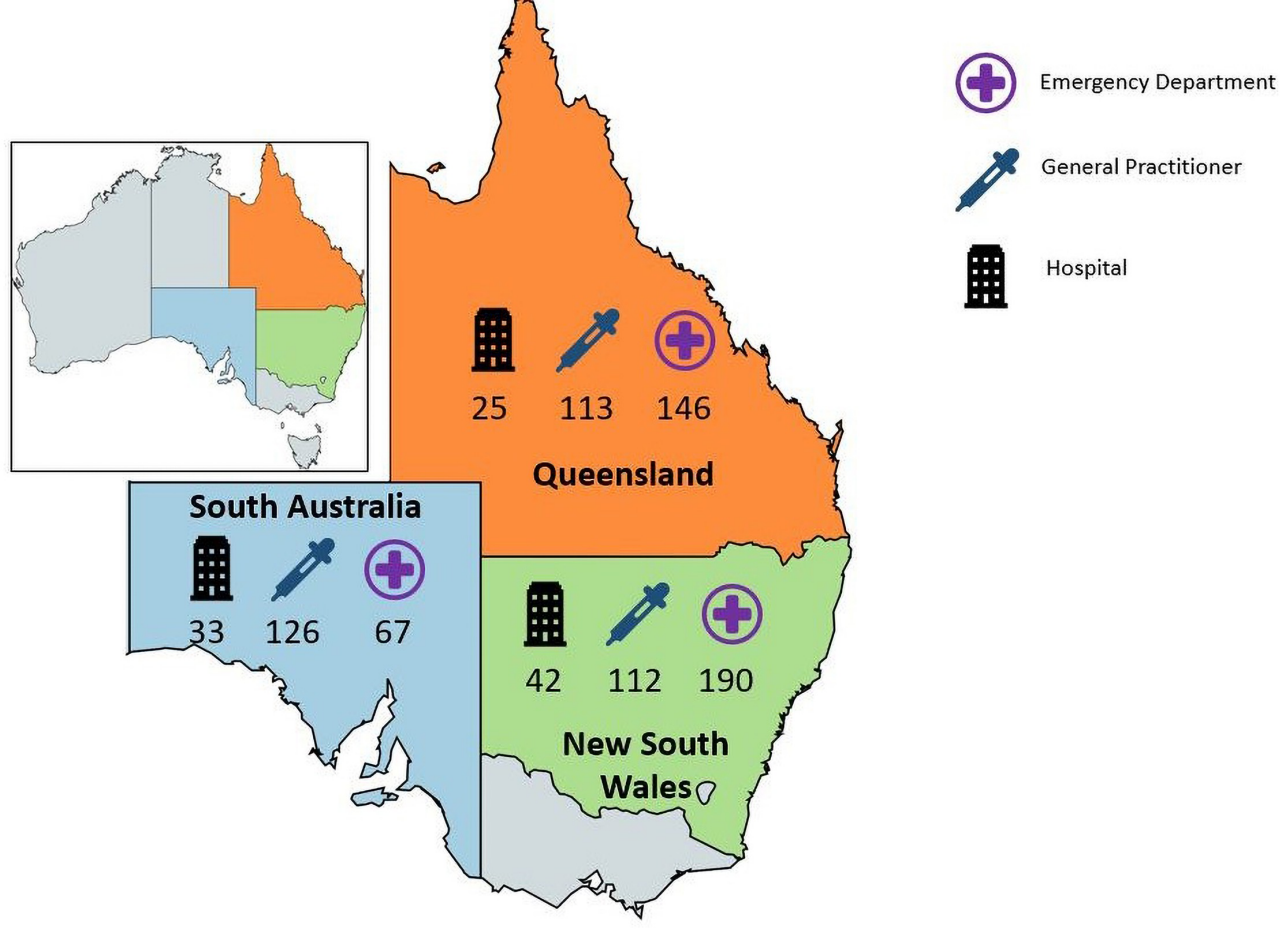
Acute Gastroenteritis assessments by state and healthcare provider type. Fig 1: total number of visits to Emergency Departments = 403; total number of admissions to hospital = 100; total number of visits to General Practitioners = 351. Total number of AGE assessments in: New South Wales = 344; Queensland = 284; and South Australia = 226. Total number of visits assessed for care of AGE in sampling frame = 854. [Adapted from https://mapchart.net/, CC BY-SA 4.0].

A minimum of 400 medical record reviews per condition was required to obtain national estimates with 95% confidence intervals (CIs) and precision of ±5%, without adjustment for design effects. Sample size calculations were based on an estimated prevalence of adherence of 0.5; this is conservative, as this is the point at which confidence intervals are widest. CTK targeted 400 medical records for AGE and 6,000 medical records for 16 other conditions. If any of the 6400 medical records targeted and sampled contained a visit for AGE, a separate assessment of adherence was made for each visit. Details of the general sampling methods have been published;[14] additional details specific to AGE can be found in S1 Appendix. Briefly, we sampled three types of health care setting (hospital inpatient admissions, ED presentations, GP consultations) in randomly-selected health department administrative units (‘health districts’) in Queensland, New South Wales and South Australia for children aged ≤15 years receiving care in 2012 and 2013 (see Fig 1). For the broader CTK study, the recruitment rate was 92% for hospitals, and estimated to be 24% for GPs (see S1 Appendix).

Medical record reviews were performed by nine experienced paediatric nurses, who were trained to assess eligibility for indicator assessment and compliance with CPGs and who were familiar with GP and hospital clinical systems. Detailed instructions were provided to the nurse surveyors (see S5 Appendix), and surveyor competence and interrater reliability was assessed on mock medical records (κ = 0.76 (95%CI, 0.75–0.77, n = 1895) for eligibility and κ = 0.71 (95%CI, 0.69–0.73, n = 1009) for compliance assessment).[14] Medical records for selected visits in 2012 and 2013 were reviewed on-site at each participating facility during March–October 2016.

The surveyor assessed each visit to determine the applicability of each indicator; if all eligibility criteria specified for the indicator were not met, the surveyor designated the indicator as ‘not applicable’ for the visit (i.e., ineligible for assessment of adherence). If all eligibility conditions were met, the surveyor recorded a ‘Yes’ (if there was documentary evidence of the compliance action being met) or ‘No’ (if no documentary evidence was present). Where ED visits ended with inpatient admission to the same hospital, the ED presentation and inpatient admission were treated as separate visits.

### Analysis

At indicator level, estimates of adherence were measured as the percentage of eligible indicators (i.e., indicators answered either ‘Yes’ or ‘No’) which were scored as ‘Yes’. At phase of care level, adherence was calculated as the proportion of all (eligible) constituent indicators that were scored as ‘Yes’.

Sampling weights were constructed to adjust for oversampling of states and healthcare settings and for sampling within health districts (see S1 Appendix)[14]. The weighted data were analysed in SAS v9.4 (SAS Institute Inc, North Carolina, USA), using the SURVEYFREQ procedure. Variance was estimated by Taylor series linearization and the primary sampling unit (health district) was specified as the clustering unit. Stratification and, where appropriate, domain analysis were used (see S1 Appendix). Exact 95% CIs were generated using the modified Clopper–Pearson method except when the point estimate was 100%, the unmodified Clopper-Pearson method was used[19]. In both indicator and phase of care reports, results were suppressed if there were <25 eligible visits to ensure patients were not identifiable. Differences in adherence rates between settings were restricted to comparisons between GP and the two hospital settings, as hospitals records were not sampled independently; similarly phases of care were not formally compared statistically as the same child usually had more than one phase of care addressed in a single visit. Statistical significance, where assessed, was based on the F-test approximation of the Rao-Scott chi-square test, which adjusts for the design effect.

### Ethical considerations

Ethics approval was gained from the Sydney Children’s Hospitals Network Human Research Ethics Committee (HREC) (HREC/14/SCHN/113), the Queensland Children’s Hospital HREC (HREC/14/QCH/91), the Women’s and Children’s Health Network HREC (HREC/14/WCHN/68), and the Royal Australian College of General Practitioners National Research and Evaluation Ethics Committee (NREEC14-008), and site-specific approvals from 34 sites were granted. Requirements for patient consent for external access to medical records was waived, as the study entailed minimal risk to providers and patients.[15] Participants were protected from litigation by gaining statutory immunity for CTK as a quality assurance activity, from the Federal Minister for Health under Part VC of the Health Insurance Act 1973 (Commonwealth of Australia).

## Results

There were 669 children with one or more eligible assessments of CPG compliance for AGE, with the age and sex distribution provided in Table 1. Over half the children (61%) in the sample were under four years of age, with roughly equal numbers of males and females.

**Table 1:** Characteristics of the eligible children, 2012–2013.

Characteristic	Children in the CTK Study
Age*	No. of children (%)
< 6 months	45 (6.7)
6–11 months	72 (10.8)
12–23 months	145 (21.7)
2–3 years	145 (21.7)
4–6 years	112 (16.7)
> 6 years	150 (22.4)
Sex	
Male	346 (51.7)
Female	323 (48.3)

*The child’s age was calculated as the age at visit where there was only one, or the midpoint of the child’s age at her first and last AGE visit, where there was more than one.

Of 30,030 possible indicator assessments for AGE, 8,010 (26.7%) were automatically excluded because they did not meet age or healthcare setting restrictions, and a further 7,586 (25.3%) were designated as not applicable or otherwise ineligible for assessment. The field team conducted 14,434 indicator assessments for AGE grouped into 854 visits, at a median of 17 indicators per visit. Eligible AGE visits were assessed in 75 GPs, 34 hospital EDs and 26 hospital inpatient service providers. Of 403 ED visits, 78 (19.4%) ended with inpatient admission to the same hospital.

### Guideline adherence

The average assessed adherence by healthcare setting is presented in Table 2, separately for each phase of care and overall. Overall adherence to guidelines by phase of care was 45.5% (95% CI: 40.7–50.4) for indicators in the ‘Diagnosis’ phase, 96.1% (95% CI: 94.8–97.1) for indicators in the ‘Treatment’ phase and 57.6% (95% CI: 51.3–63.7) in the ‘Ongoing Management’ phase.

**Table 2:** Adherence to care, 2012–2013.

Phase of Care	Healthcare Setting	No. of Children	No. of Visits	No. of Indicators Assessed	Proportion Adherent % (95% CI)
Diagnosis	GP	312	351	3199	38.5 (33.7, 43.4)
	ED	342	403	3722	85.7 (82.4, 88.7)
	Inpatient	94	100	923	87.1 (80.2, 92.3)
	Overall	669	854	7844	45.5 (40.7, 50.4)
Treatment	GP	308	346	1246	96.9 (94.9, 98.3)
	ED	337	395	1648	91.3 (88.2, 93.8)
	Inpatient	79	85	284	94.7 (89.7, 97.7)
	Overall	656	826	3178	96.1 (94.8, 97.1)
Ongoing management	GP	290	325	957	51.6 (40.3, 62.8)
	ED	309	360	1864	77.2 (74.2, 80.0)
	Inpatient	94	100	591	74.9 (66.4, 82.2)
	Overall	635	785	3412	57.6 (51.3, 63.7)
All phases of care	GP	312	351	5402	54.6 (51.1, 58.1)
	ED	342	403	7234	84.7 (82.4, 86.9)
	Inpatient	94	100	1798	84.3 (80.0, 87.9)
	Overall	669	854	14 434	59.6 (56.7–62.5)

GP = General Practice; ED = Emergency Department.

Adherence to guidelines overall was significantly higher for both ED (84.7%; 95% CI: 82.4–86.9) and hospital inpatients (84.3%; 95% CI: 80.0–87.9) than for GPs (54.6%; 95% CI: 51.1–58.1; p<0.0001 for both comparisons). Adherence in the ‘Diagnosis’ phase of care varied by healthcare setting, being lower in the GP setting (38.5%; 95% CI: 33.7–43.4) than the ED (85.7%; 95% CI: 82.4–88.7) and hospital inpatient (87.1%; 95% CI: 80.2–92.3) settings; p<0.0001 for both comparisons. Adherence in the ‘Treatment’ phase of care was high (>90%) in all settings. For ‘Ongoing management’, adherence was again lower in the GP setting (51.6%; 95% CI: 40.3–62.8) than in either inpatients (74.9%; 95% CI: 66.4–82.2; p = 0.003) or ED settings (77.2%; 95% CI: 74.2–80.0; p = 0.002).

The assessed adherence for individual indicators by healthcare setting is shown in Table 3. Adherence was not reported where there were fewer than 25 visits where an indicator was assessed. For the indicators where adherence was assessed, compliance ranged from 5% for indicator AGE09 (“*Children who presented with gastroenteritis had their observations (*temperature, heart rate, respiratory rate and blood pressure) *assessed”*) in GPs, to 100% for indicators AGE04 (*“Children who presented with gastroenteritis had the duration of their illness recorded”*) in ED and AGE20 (“*Children with gastroenteritis and no signs of infection were not prescribed anti-diarrheals (such as loperimide*, *kaolin*)”) in inpatient settings.

**Table 3:** Adherence of care, by clinical indicator, 2012–2013.

Indicator ID	Indicator Description	Care Setting	No. of Children	No. of Visits	Proportion Adherent, % (95% CI)
AGE01	Children who presented with gastroenteritis had their fluid intake recorded.	GP	312	351	35.8 (28.0, 44.1)
		ED	342	403	87.8 (81.4, 92.6)
		Inpatient	94	100	96.3 (78.1, 100.0)
		Overall	669	854	43.6 (35.3, 52.2)
AGE02	Children who presented with gastroenteritis had their urine output recorded.	GP	312	351	14.2 (9.2, 20.7)
		ED	342	403	78.7 (70.3, 85.6)
		Inpatient	94	100	95.2 (82.3, 99.5)
		Overall	669	854	24.1 (18.0, 31.1)
AGE03	Children who presented with gastroenteritis had the frequency of their vomiting and diarrhea recorded.	GP	312	351	65.2 (50.9, 77.8)
		ED	342	403	95.2 (91.6, 97.6)
		Inpatient	94	100	88.8 (47.2, 99.8)
		Overall	669	854	69.5 (57.9, 79.6)
AGE04	Children who presented with gastroenteritis had the duration of their illness recorded.	GP	312	351	79.2 (65.0, 89.5)
		ED	342	403	100.0 (99.1, 100.0)
		Inpatient	94	100	88.3 (48.2, 99.8)
		Overall	669	854	82.0 (70.5, 90.4)
AGE05	Children who presented with gastroenteritis had their weight recorded.	GP	312	351	20.6 (9.0, 37.3)
		ED	342	403	91.7 (86.9, 95.1)
		Inpatient	94	100	97.2 (90.1, 99.7)
		Overall	669	854	31.2 (20.6, 43.6)
AGE06	Children who presented with gastroenteritis were assessed for lethargy.	GP	312	351	43.3 (27.8, 59.9)
		ED	342	402	95.2 (91.8, 97.5)
		Inpatient	94	100	98.9 (93.2, 100.0)
		Overall	669	853	51.0 (36.8, 65.1)
AGE07	Children who presented with gastroenteritis had their mucous membranes assessed.	GP	312	351	32.3 (18.1, 49.4)
		ED	342	403	70.4 (56.9, 81.7)
		Inpatient	94	100	60.9 (39.2, 79.8)
		Overall	669	854	37.7 (27.2, 49.1)
AGE08	Babies (aged < 12 months) who presented with gastroenteritis had their fontanelle assessed.	GP	37	40	15.7 (2.2, 44.9)
		ED	83	99	55.8 (36.9, 73.5)
		Inpatient	24	24	Insufficient data
		Overall	123	163	27.4 (14.8, 43.3)
AGE09	Children who presented with gastroenteritis had their observations (Temp, HR, RR, BP) assessed.	GP	312	351	5.0 (3.0, 7.9)
		ED	341	402	85.1 (76.5, 91.5)
		Inpatient	94	100	83.2 (54.4, 97.3)
		Overall	668	853	16.8 (13.1, 21.1)
AGE10	Children who presented with gastroenteritis had their degree of dehydration assessed.	GP	312	351	53.0 (39.0, 66.7)
		ED	341	401	74.5 (66.3, 81.7)
		Inpatient	93	99	79.5 (63.6, 90.7)
		Overall	669	851	56.3 (46.1, 66.0)
AGE11	Children who presented to the ED with gastroenteritis and required intravenous therapy, received electrolytes.	ED	85	91	83.5 (65.2, 94.6)
		Overall	85	91	83.5 (65.2, 94.6)
AGE12	Children who presented to the ED with gastroenteritis and required intravenous therapy, received a venous blood gas.	ED	87	93	61.2 (45.5, 75.3)
		Overall	87	93	61.2 (45.5, 75.3)
AGE13	Children who presented to the ED with gastroenteritis and severe dehydration, received electrolytes.	ED	17	17	Insufficient data
		Overall	17	17	Insufficient data
AGE14	Children who presented to the ED with gastroenteritis and severe dehydration, received a venous blood gas.	ED	17	17	Insufficient data
		Overall	17	17	Insufficient data
AGE15	Children who presented to the ED with gastroenteritis and altered conscious state/convulsions received electrolytes.	ED	8	9	Insufficient data
		Overall	8	9	Insufficient data
AGE16	Children who presented to the ED with gastroenteritis and altered conscious state/convulsions received a venous blood gas.	ED	7	8	Insufficient data
		Overall	7	8	Insufficient data
AGE17	Children who presented to the ED with gastroenteritis and pre-existing medical conditions that predispose to electrolyte abnormalities (e.g. cystic fibrosis, renal impairment, diabetes), received electrolytes.	ED	25	28	77.1 (57.4, 90.7)
		Overall	25	28	77.1 (57.4, 90.7)
AGE18	Children who presented to the ED with gastroenteritis and pre-existing medical conditions that predispose to electrolyte abnormalities (e.g. cystic fibrosis, renal impairment, diabetes), received a venous blood gas.	ED	25	28	55.9 (36.0, 74.5)
		Overall	25	28	55.9 (36.0, 74.5)
AGE19	Children with gastroenteritis and NO signs and symptoms of dehydration, did not receive routine blood tests.	GP	273	304	99.6 (98.1, 100.0)
		ED	209	240	96.8 (93.7, 98.7)
		Inpatient	33	34	94.7 (81.1, 99.4)
		Overall	496	578	99.4 (98.3, 99.8)
AGE20	Children with gastroenteritis and no signs of infection (were not prescribed anti-diarrheals (such as loperimide, kaolin).	GP	281	315	96.7 (91.3, 99.2)
		ED	313	368	99.8 (98.7, 100.0)
		Inpatient	72	78	100.0 (95.4, 100.0)
		Overall	608	761	97.1 (92.8, 99.2)
AGE21	Children with gastroenteritis and no signs of infection were not prescribed maxalon, stemetil, multi-dose ondansetron.	GP	280	313	93.8 (88.6, 97.1)
		ED	313	369	88.2 (76.6, 95.4)
		Inpatient	74	80	90.8 (71.6, 98.7)
		Overall	607	762	93.1 (89.2, 95.9)
AGE22	Children with gastroenteritis and no signs of infection were not prescribed antibiotics.	GP	280	314	97.6 (94.6, 99.2)
		ED	307	363	99.1 (97.4, 99.8)
		Inpatient	74	80	98.9 (93.4, 100.0)
		Overall	604	757	97.8 (95.6, 99.1)
AGE23	Children who presented with gastroenteritis and were severely dehydrated, received IV fluid rehydration including a 20 ml/kg bolus.	ED	17	17	Insufficient data
		Inpatient	12	12	Insufficient data
		Overall	23	29	79.3 (51.6, 95.2)
AGE24	Children who presented with gastroenteritis, had no or mild signs of dehydration, and were able to tolerate oral fluids were discharged from hospital.	ED	233	274	93.6 (89.4, 96.4)
		Inpatient	45	48	75.2 (30.7, 97.8)
		Overall	261	322	91.0 (83.8, 95.8)
AGE25	Children who presented with gastroenteritis, had no or mild signs of dehydration, and were able to tolerate oral fluids were advised to re-present if symptoms are unchanged or worsen.	GP	280	312	64.3 (55.3, 72.7)
		ED	229	270	82.0 (76.2, 87.0)
		Inpatient	45	48	42.8 (18.7, 69.7)
		Overall	541	630	65.7 (58.4, 72.4)
AGE26	Children who presented with gastroenteritis, had no or mild signs of dehydration, and were able to tolerate oral fluids were advised to continue with usual diet.	GP	283	315	28.4 (14.7, 45.8)
		ED	234	275	53.0 (42.5, 63.4)
		Inpatient	47	50	58.1 (23.5, 87.6)
		Overall	547	640	31.1 (20.5, 43.5)
AGE27	Children who presented with gastroenteritis, had no or mild signs of dehydration, and were able to tolerate oral fluids were provided with information on age-appropriate oral fluid replacement (small fluids often; breastfeeding/formula, oral rehydration solution or dilute clear fluids).	GP	284	317	61.4 (43.5, 77.3)
		ED	235	276	67.2 (56.3, 76.9)
		Inpatient	47	50	64.7 (43.0, 82.8)
		Overall	549	643	62.0 (47.9, 74.8)
AGE28	Children who presented to the GP with gastroenteritis and moderate or severe dehydration were referred to hospital or the ED.	GP	13	13	Insufficient data
		Overall	13	13	Insufficient data
AGE29	Children who presented with gastroenteritis, were moderately to severely dehydrated AND received rehydration, had their weight reassessed within 6 hours.	ED	66	68	12.1 (1.8, 35.5)
		Inpatient	40	41	17.8 (6.3, 36.2)
		Overall	82	109	13.3 (4.2, 29.2)
AGE30	Children who presented with gastroenteritis, were moderately to severely dehydrated AND received rehydration, were reassessed for clinical signs of dehydration within 6 hours.	ED	66	68	92.0 (82.9, 97.2)
		Inpatient	40	41	82.7 (54.8, 96.9)
		Overall	82	109	90.0 (81.1, 95.7)
AGE31	Children who presented with gastroenteritis, were moderately to severely dehydrated AND received rehydration, had their urine output reassessed within 6 hours.	ED	67	69	90.4 (78.4, 97.0)
		Inpatient	41	42	93.7 (78.1, 99.3)
		Overall	84	111	91.1 (82.6, 96.3)
AGE32	Children who presented with gastroenteritis, were moderately to severely dehydrated AND received rehydration, were reassessed for ongoing diarrhea/vomiting within 6 hours.	ED	67	69	96.3 (85.4, 99.7)
		Inpatient	41	42	94.2 (77.5, 99.6)
		Overall	84	111	95.9 (88.1, 99.2)
AGE33	Children who presented with gastroenteritis, were moderately to severely dehydrated AND received rehydration, were reassessed for signs of fluid overload (puffy face and extremities) within 6 hours.	ED	66	68	47.3 (25.8, 69.5)
		Inpatient	40	41	46.4 (17.5, 77.3)
		Overall	82	109	47.1 (29.9, 64.7)
AGE34	Children with gastroenteritis who were sufficiently rehydrated as indicated by weight gain and/or clinical status (child is rehydrated or only mildly dehydrated) were discharged.	ED	171	195	93.2 (88.3, 96.6)
		Inpatient	87	93	98.4 (93.3, 99.9)
		Overall	238	288	94.5 (90.9, 97.0)
AGE35	Children with gastroenteritis who had gastrointestinal loss that was not profuse (oral intake equals or exceeds losses), were discharged.	ED	197	232	95.6 (91.7, 98.0)
		Inpatient	89	95	98.4 (93.5, 99.9)
		Overall	267	327	96.2 (93.3, 98.1)

GP = General Practice; ED = Emergency Department; HR = Heart Rate; Temp = Temperature; RR = Respiratory Rate; BP = Blood Pressure.

Some of the indicators with the lowest compliance related to the assessment and documentation of children presenting with AGE. Overall, only 16.8% (95% CI: 13.1–21.1) of children had their observations recorded (AGE09), due to very low documentation among GPs (5.0%; 95% CI: 3.0–7.9). Similarly, when presenting to GPs, only 14.2% of children had their urine output recorded (AGE02; 95% CI: 9.2–20.7) and less than a quarter had their weight recorded (AGE05; 20.6%; 95% CI: 9.0–37.3). Across all settings, babies under one-year old presenting with AGE had their fontanelles assessed and recorded (depression of the anterior fontanelle is a clinical sign of dehydration in infants) only 27.4% of the time (AGE08; 95% CI: 14.8–43.3). Families of children with AGE, who are able to tolerate oral fluids, were documented to have received appropriate advice to continue with usual diet about one third of the time (AGE26; 31.1%; 95% CI: 20.5–43.5).

There was high adherence to guidelines discouraging overuse of anti-diarrheals (AGE20; 97.1%; 95% CI: 92.8–99.2), anti-emetics (AGE21; 93.1%; 95% CI: 89.2–95.9), antibiotics (AGE22; 97.8%; 95% CI: 95.6–99.1), and routine blood tests (AGE19; 99.4%; 95% CI: 98.3–99.8).

## Discussion

According to retrospective chart review, participants received about 60% of recommended processes in their care for AGE; more so if they attended EDs and specialist paediatricians. This proportion of guideline-adherent documented care (59.6%; 95%CI 56.7–62.5%) is very similar to the findings across all 17 combined common childhood conditions in the CTK study (59.8%; 95%CI 57.5–62.0%)[14]. For all settings, documented compliance with individual AGE indicators ranged from 13.3% to 99.4%; documented compliance with guidelines varied by phase of care, being highest in the treatment phase and lowest in the diagnosis phase.

Many appropriate initial assessments were not documented when children presented to GPs with AGE, such as the duration of illness, frequency of symptoms, and routine clinical observations (temperature, heart rate, respiratory rate, blood pressure). It is not clear how much of this is because children were not adequately assessed, versus poor documentation of assessments. This inability to distinguish between poor clinical practice and poor documentation is a common problem with retrospective record review research, and highlights the need for other research methods to be used to assist in the interpretation of the findings Patients presenting at GPs are likely to have milder symptoms than those presenting to hospital, so excluding some recommended observations (e.g. blood pressure) may be clinically justifiable. Quality improvement programs that rely on medical records for evidence of compliance of appropriate care may prompt improved documented compliance with quality indicators that do not actually improve the care received[20]. However, the indicators selected for review in the CTK study were rated by an expert panel for their clinical impact and thus there is a reasonable expectation that they should be undertaken and documented. For example, failing to explicitly document certain initial assessments (e.g. only 20% of GP presentations had a patient weight recorded) makes assessment of a deteriorating patient’s clinical condition difficult. Moreover, lack of documented initial assessments is concerning given the importance of the medical record as a clinical and legal document, and it is generally accepted that good documentation is an integral component of high quality health care[21].

A positive finding is that children presenting with AGE to GPs and hospitals are generally not receiving unnecessary treatment or tests: overall, few anti-diarrheals, anti-emetics, antibiotics, or blood tests (AGE19-AGE22) were ordered. Anti-diarrheals such as loperamide are not recommended for use in children as the benefit of a slight reduction in duration of symptoms (0.8 days, on average) is outweighed by the risk of serious side effects such as ileus[6]. Testing for the pathogenic causes of gastroenteritis (e.g., bacterial vs viral) is expensive and does not usually influence treatment[22], although it may occasionally be indicated for public health surveillance and prevention and control measures. The majority of AGE cases are caused by viral pathogens, and the use of antibiotics is not routinely recommended even in cases with a suspected bacterial aetiology; as the majority of cases of AGE are self-limiting and not shortened by antibiotic treatment[23].

Only one third of families of children with AGE who were able to tolerate oral fluids, were documented to have received appropriate advice to continue with usual diet. Our results are consistent with other studies from the US and Europe which have shown clinicians’ dietary management of children with AGE in practice to be at odds with CPGs for several decades[24, 25]. As with initial assessments, it is possible that appropriate advice is being given to the carer but not documented in the medical record, as advice is much less likely to be recorded than other aspects of care (e.g. medications prescribed)[26]. Another possibility is that advice to make simple dietary changes may be used by clinicians as a means of reassuring families who have an expectation of some form of ‘treatment’. Despite the established recommendations for early reintroduction of normal diet, a recent systematic review suggests that there is no clear advantage of early vs late re-feeding[27], and so deviation from guidelines may not be clinically significant.

This study highlights room for improvement in CPG adherent care for AGE in Australia, particularly in the primary care setting. Several interventions have been trialled to increase physician compliance with AGE CPGs, and demonstrate that reasonably simple education programs may be effective in improving the quality of care for this condition. For example, an eLearning course completed by physicians in 11 European countries was effective in increasing knowledge scores, average adherence, and the number of patients treated in full accordance with the guidelines[28]. A randomized controlled trial of Italian primary care physicians reported that paediatricians who completed a two-hour training course on AGE management treated children in full accordance with the guidelines 65.5% of the time, compared with only 3% in the control group[9]. Children who were treated according to the guidelines had a shorter duration of illness and a small but statistically significant increase in weight gain compared with children in the control group[9], highlighting the value of CPG adherence. Other hospital-based interventions have been demonstrated to improve adherence with oral rehydration guidelines, reducing the use of higher risk and more expensive IV hydration[29–31].

One limitation of the current study, and a factor which may be contributing to variance in the proportion of guideline-adherent care, is the lack of Australia-wide consensus on guidelines for the treatment of children with AGE, which limits the interpretation of the proportion adherent for some of the indicators reported. There is some variance in the CPGs promoted for the treatment of AGE in children between states, health districts and hospitals. For example, the CPG for Gastroenteritis for the Royal Children’s Hospital in Melbourne, recommended for use state-wide in Victoria, recommends paediatric patients undergoing rehydration for AGE are re-weighed 6-hourly using bare weight[22]. However the New South Wales Health CPG does not require re-weighing[32]. This variation in recommendations may account for some proportion of the non-adherent behaviour, but it should be noted that external reviewers concluded that the Victorian CPG was the appropriate standard of care for assessing clinical practice in Australia in 2012 and 2013[16]. Nevertheless, as sampling was conducted in only three states, the generalisability of the results to the other three states and two territories is uncertain. The adoption of consistent national (or international) clinical guidelines for the management of AGE would provide clarity to clinicians, in addition to making it easier to evaluate quality of care.

The clinical indicators were based on CPG recommendations relevant for the years 2012–2013, and were developed by researchers and clinicians in an Australian setting. While this limits the generalisability and replicability of our study findings beyond these contexts, our indicator set and overall methodological approach form a basis from which new indicators may be derived and benchmarking activities adapted to local settings[18]. We did not critically appraise the methodological quality of included CPGs; grades of recommendations and levels of evidence were recorded verbatim from published CPGs. As a reflection of the CPGs from which they were born, all indicators were based on consensus-level recommendations.

Another limitation of the study is that, despite the large sample, we did not collect sufficient encounters related to rarer presentations (e.g., children who presented to ED with gastroenteritis and severe dehydration, AGE13-AGE14, or altered consciousness/convulsions, AGE14-15), and we cannot therefore be confident of the estimated rate of adherence. As a retrospective audit, the study was not able to assess whether guideline-adherent care was provided but not documented; further studies are required to determine whether assessment, re-assessment, treatment or advice is being provided in accord with clinical guidelines but not documented. Self-selection of GPs, and the low estimated recruitment rate (24%), could lead to bias in the estimated adherence in GPs (see S1 Appendix).

## Conclusion

This study estimates, for the first time, the guideline-adherence of care provided to children presenting with AGE, across multiple treatment settings in multiple states of Australia. Although the proportion of children receiving appropriate therapies (e.g. medications, fluids, electrolytes) during the treatment phase of care is high (>90%), it demonstrates that some other elements of care, including assessment, documentation (including the child’s weight), and advice may be provided in only a minority of cases, with variation by clinical setting. Further work is required to determine the best way to improve compliance with recommended diagnostic assessment and address a lack of comprehensive care documentation.

## Supporting information

S1 Appendix Additional details relating to study methods. (DOCX)

S2 Appendix Excluded AGE candidate indicators. (DOCX)

S3 Appendix Conversion of AGE candidate indicators to medical record indicator questions. (DOCX)

S4 Appendix Key information from the CareTrack Kids Surveyors’ Manual relevant to assessment of AGE indicators. (DOCX)

S1 Table Characteristics of final clinical indicators for Acute Gastroenteritis. (DOCX)

## Abbreviations

AGE: Acute Gastroenteritis
CI/s: Confidence interval/s
CPG: clinical practice guideline
CTK: CareTrack Kids
ED: Emergency Department
ESPGHAN: European Society for Paediatric Gastroenterology, Hepatology, and Nutrition
GP: general practitioner (family physician)
NICE: National Institute for Health and Clinical Excellence
