# Supplementary material for: Appropriate management of acute gastroenteritis in Australian children: A population-based study
Source: PLoS One. 2019 Nov 7;14(11):e0224681. doi: 10.1371/journal.pone.0224681 (PMC6837505; doi:10.1371/journal.pone.0224681)
Supplement: S1 Appendix — (DOCX) [file pone.0224681.s001.docx]

**S1 Appendix: Additional details relating to study methods**

The report of top-level CareTrack Kids (CTK) results^1^ and its associated online appendix detail the methods of the larger study, which generated the data reported in this paper. Selected methods specifically relevant to acute gastroenteritis are described below.

**Sample size**

A visit was defined as an occasion of admitted care for inpatients, a presentation for Emergency Department (ED) care or a consultation with a general practitioner. Without adjustment for the design effect, a minimum of 400 visits per condition was required to obtain national estimates with 95% Confidence Interval (CI) and precision of +/- 5% at condition level, conservatively assuming only one assessable indicator per visit. It was anticipated that loss of precision due to design effects would be largely offset by multiple assessable indicators per visit and additional visits generated by secondary sampling (multiple visits for care of acute gastroenteritis for each medical record identified for sampling of acute gastroenteritis, and visits for care of acute gastroenteritis incidentally found in medical records identified for sampling other conditions).

**Sampling** **Process**

A multistage stratified random sampling process was implemented. For logistical efficiency, sampling was targeted at three states, Queensland (QLD), New South Wales (NSW) and South Australia (SA), which together comprise 60.0% of the estimated Australian population aged 15 years or younger in the 2012 and 2013 calendar years. All six paediatric tertiary hospitals (two in QLD, three in NSW, and one in SA) were targeted as they have state-wide coverage. State Departments of Health organize care within administrative units (‘health districts’): Hospital Health Services in QLD, Local Health Districts in NSW, and Local Health Networks in SA. For QLD, we targeted five health districts (two metropolitan, three regional), in NSW four health districts (two metropolitan, two regional), and in SA three health districts (two metropolitan, one regional).

**Recruitment of health care providers**

Within the selected health districts, we approached all public hospitals, or private hospitals providing public services under contract, that had patient volumes of ≥2,000 ED presentations and ≥500 paediatric separations per year; we also advertised the study to General Practices (GPs), and approached all the providers we could identify through internet searches, and via personal contacts. Within the selected sites, we sampled medical records for each condition targeted at that setting.

As noted in the main text, 34 of 37 (92%) eligible hospitals that were approached agreed to participate. Recruitment of GPs was decentralized. Administrative details for refusal rates, from cold-calling or direct contact by clinicians who facilitated recruitment of their peers, were maintained on project laptops. At the end of recruitment all computers were decommissioned and cleaned, with the files archived on a USB. Unfortunately, the USBs created during laptop decommissioning were misplaced and have not been able to be located. This did not affect the indicator adherence data, as the database was remotely located and updated regularly via the internet. We have therefore sought to estimate the recruitment rates based on recruitment spreadsheets emailed to the administrative staff.

We were only able to locate emailed spreadsheets with late stage records for one state, South Australia. Based on this spreadsheet, we approached 114 GPs and recruited 27 of them, giving a recruitment rate of 23.7%; an additional GP, not listed on the available spreadsheet, was recruited subsequently and was not added to either the numerator or the denominator, for this estimate. The spreadsheet did not have clear information on eligibility, so it is likely that an unknown number of the 114 approached were ineligible because: 1) they were not open during the whole 2012-2013 survey period; 2) they saw no or few children; or 3) they were not confident in their ability to generate full listings of children with the target conditions, or they did not use one of the four practice software systems our surveyors were trained to search. Our estimate of 23.7% is therefore likely to be an underestimate of the actual recruitment rate.

Self-selection of GPs, and the estimated 24% recruitment rate, could lead to bias in the estimated guideline adherence, arising from self-selection. It is plausible that self-selected practices were more confident of their guideline adherence, potentially leading to overestimation of the quality of care in the CareTrack Kids study.

**Allocation of target samples to sampling units and sampling process**

The number of acute gastroenteritis records targeted at each site was determined by a nominal allocation of the 400 records targeted, informed by data available at the time, supplemented by expert opinion, with planned over-sampling of settings where fewer occasions of care were expected.^1,2^ For hospitals, a fixed number was targeted at each site, for each setting; for GPs, different combinations of conditions were targeted at each site, to simplify the logistics of sampling.

Hospitals identified all inpatient visits for AGE with the ICD-10 codes A08.x or A09.x in 2012 and 2013. EDs in South Australia and Queensland also used this ICD-10 code while in New South Wales, the SNOMED code 69776003 was used. GPs used their local systems to identify all visits for AGE. Within each site, the lists of visits were ordered randomly and sampled sequentially until the allocated quota for the site was met.

If a record sampled for AGE contained multiple visits for management of AGE, each visit was assessed separately. If a medical record sampled for a condition other than AGE contained a visit where AGE was managed, those visits were also assessed. Visits identified through secondary sampling were only included if they indicated a clear diagnosis of AGE.

**Data collection**

Nine experienced paediatric nurses were employed across the three states, with all nine assessing occasions of care for acute gastroenteritis. The surveyors undertook a one-week training program, prior to data collection. A surveyor manual was developed which included instructions, condition-specific definitions, inclusion and exclusion criteria, and guidance for assessing eligibility of each encounter for relevant indicators.

A web-based tool, originally developed for the CareTrack Adults study^3,4^, was designed to enter data during medical record review. Algorithms to filter indicators by setting, and by age, were embedded in the tool. For acute gastroenteritis, there was one age-specific filter (AGE08, restricted to children < 1 year of age), and multiple setting filters (one restricted to GPs, nine restricted to ED presentations and inpatients, and eight restricted to ED presentations alone, leaving 17 unrestricted).

Surveyors undertook criterion-based medical record reviews using the data collection tool. Surveyors assessed the record for evidence that the participant presented for management of acute gastroenteritis in the years 2012 and 2013. The surveyors responded to each indicator as ‘Yes’ (care provided during the encounter was consistent with the indicator), ‘No’, or ‘Not Applicable’ (NA; the indicator was not eligible for assessment). For example, a surveyor assessing an occasion of care for a mildly dehydrated child would record ‘NA’ to indicators AGE29-AGE33 which ask about children who are moderately or severely dehydrated.

**Analysis**

Survey or register-derived data were used to estimate the proportion of occasions of care for acute gastroenteritis.^5-10^ The number of occasions of healthcare for each condition was thereby estimated for each hospital (inpatient and ED) or each health district (GP), and sampling weights were calculated using the methods detailed in eAppendix 4 of the broader CTK study (this Appendix can be accessed by request via the corresponding author, if required).^1^

A variety of stratifications, and sometimes domain analysis,^11,12^ were necessary to ensure accuracy of the confidence interval estimates. These are detailed in eTable 1, below.

**eTable 1: Domain analysis and stratifications for different estimates presented in the manuscript.**

| Location | Sub-section/Area | Domain analysis^11,12^ | Strata |
| --- | --- | --- | --- |
| Table 2 | Phase of care x healthcare setting estimates | Yes | State |
|  | Estimate for phase of care (all settings) | Yes | State and healthcare setting |
|  | Estimate for healthcare setting (all phases) | None | State |
|  | Overall estimate for all phases and settings* | None | State and healthcare setting |
| Table 3 | Indicator x healthcare setting estimates | Yes | State |
|  | Overall indicator estimates | Yes | State and healthcare setting |

* This estimate was previously published in the top-level results for the broader CTK study.^1^

**References:**

1. Braithwaite J, Hibbert PD, Jaffe A, et al. Quality of health care for children in Australia, 2012-2013. *JAMA*. 2018;319(11):1113-1124.

2. Hooper TD, Hibbert PD, Mealing N, et al. CareTrack Kids-part 2. Assessing the appropriateness of the healthcare delivered to Australian children: study protocol for a retrospective medical record review. *BMJ Open*. 2015;5(4):e007749.

3. Hunt TD, Ramanathan SA, Hannaford NA, et al. CareTrack Australia: assessing the appropriateness of adult healthcare: protocol for a retrospective medical record review. *BMJ Open*. 2012;2(1):e000665.

4. Runciman WB, Hunt TD, Hannaford NA, et al. CareTrack: assessing the appropriateness of health care delivery in Australia. *Med J Aust*. 2012;197(2):100-105.

5. Britt H, Miller GC, Henderson J, et al. *General Practice Activity in Australia 2012-13: BEACH: Bettering the Evaluation and Care of Health*. Sydney, Australia: Sydney University Press; 2013.

6. Harrison C. BEACH 2012-13 weighted data on frequency of management of selected conditions, for children aged 0-15, by General Practitioners. [Personal communication] In. Sydney, Australia: Menzies Centre for Health Policy, School of Public Health, The University of Sydney; 2017.

7. Australian Institute of Health and Welfare. *Australian hospital statistics 2012–13: Emergency department care*. Vol 52. Canberra, Australia: AIHW; 2013.

8. Queensland Health, New South Wales Health, South Australian Department of Health. Emergency Department data on frequency of management of selected conditions, for children aged 0-15. [Personal communication] In:2017.

9. Australian Institute of Health and Welfare. *Australian hospital statistics 2012–13*. Vol 54. Canberra, Australia: AIHW; 2014.

10. Australian Institute of Health and Welfare. Inpatient separations for selected conditions, as identified by ICD-10 principal diagnoses. Available at: <http://www.aihw.gov.au/hospitals-data/principal-diagnosis-data-cubes/>. Accessed 2017

11. Lohr S. *Sampling: design and analysis*. Second ed. Boston, MA: Brooks-Cole Publishing; 2009.

12. Heeringa SG, West BT, Berglund PA. *Applied survey data analysis*. Boca Raton, FL: CRC Press; 2010.
