# Supplementary material for: Appropriate management of acute gastroenteritis in Australian children: A population-based study
Source: PLoS One. 2019 Nov 7;14(11):e0224681. doi: 10.1371/journal.pone.0224681 (PMC6837505; doi:10.1371/journal.pone.0224681)
Supplement: S2 Appendix — (DOCX) [file pone.0224681.s002.docx]

**S2 Appendix. Excluded AGE candidate indicators.**

The rejected clinical indicators are presented for ‘Acute gastroenteritis’ (AGE), with their source, level of evidence, phase of care, and stage (of the indicator development process) at which and reasons why they were excluded.

| **Condition** | **Rejected indicator** | **Source** | **Level of evidence** | **Phase of care** | | | | | **Stage of development process excluded** | | | | | | **Reason(s) for exclusion** | | | | | | **Comment(s)** |  |
| --- | --- | --- | --- | --- | --- | --- | --- | --- | --- | --- | --- | --- | --- | --- | --- | --- | --- | --- | --- | --- | --- | --- |
|  |  |  |  | **Diagnosis** | **Treatment** | **Ongoing management** | **Screening** | **Internal review Round 1** | | **Internal review Round 2** | **Internal review Round 3** | **External review (wiki) Round 1** | **External review (wiki) Round 2** | **Post-wiki** | | **Acceptability** | **Feasibility** | **Impact** | **Covered in other indicator(s)** | **Low appropriateness score (<7)** |  | |
| AGE | Children presenting with gastroenteritis should NOT be prescribed Ondansetron if aged <6months or weigh <8kg | RCH Melb 2013a | consensus |  | ✔ |  |  | ✔ | |  |  |  |  |  | |  |  | ✔ | ✔ |  | Covered in another indicator (Ondansetron is not recommended in GP under age 2 years)  Too rare | |
| AGE | Children presenting with gastroenteritis may be treated with probiotics if used in conjunction with an Oral Rehydrating Solution (ORS) | Churgay 2012 | consensus |  | ✔ |  |  | ✔ | |  |  |  |  |  | | ✔ |  |  |  |  | “may be” wording  A Cochrane review of 63 studies concluded that probiotics reduce the duration of diarrhoea by approx. one day when used in conjunction with an ORS.  Evidence unclear. | |
| AGE | Children with gastro who have no/mild dehydration presenting to the emergency department should be discharged home following:  - a trial of oral fluids (10-20ml over 1 hour), which has been retained  - clinical signs of dehydration have improved  - has passed urine  - parents understand the importance of oral rehydration and are able to continue this at home  - appropriate follow-up arranged e.g. review with GP next day  - parents advised that a few small vomits does not mean the trial has failed | RCH Melb 2013a | consensus |  | ✔ |  |  |  | | ✔ |  |  |  |  | | ✔ | ✔ |  |  |  | Too complex  Trial of fluids not mandatory if no or minimal dehydration | |
| AGE | Children with gastroenteritis who are being managed with IV fluids should continue (as tolerated) their age appropriate diet and fluids. | Cincinnati 2011 | consensus |  | ✔ |  |  |  | | ✔ |  |  |  |  | |  | ✔ |  |  |  | No Australian primary source.  Unlikely to be documented. | |
| AGE | Children presenting to the emergency department with gastroenteritis and moderate dehydration should be rehydrated in order of preference with progression the next stage if indicated:  1. “Aggressive” and diligent oral rehydration (breastfeeding, ORS), reassess after 2hrs and consider the next step if ongoing losses continue  2a. RAPID NG ORS rehydration 25ml/kg/hr for 4 hrs (max 300ml) eg Gastrolyte®, Hydralyte®, Pedialyte®  - if vomiting continues once NG rehydration has commenced consider administration of ondansetron and slow NG fluids temporarily  - Ondansetron wafer dose: 8-15kg = 2mg, 15-30kg =4mg, >30kg =8mg  2b. SLOWER NG (SEE DEFINITIONS) rehydration should occur if: infants <6mths, presence of comorbidities, significant abdominal pain and consists or replacing the deficit over 6hrs (10ml/kg/hr) and then daily maintenance over the next 18hrs (dependent on weight).  Progress to IV fluids (severe dehydration management) if :  - vomiting continues on NG rehydration (despite halving the rate)  - profuse ongoing diarrhoea | NSW Health 2010 | consensus |  |  |  |  |  | | ✔ |  |  |  |  | |  | ✔ |  |  |  | Too complex to measure (cascading actions). | |
| AGE | Children presenting with gastroenteritis and no/mild clinical signs of dehydration should receive parental advice about planned follow-up and review if there is a failure to improve, deterioration or development of new signs | NSW Health 2010 | consensus |  |  | ✔ |  | ✔ | |  |  |  |  |  | |  | ✔ | ✔ |  |  | Unlikely to be documented. Low impact. | |
| AGE | Infants should be immunised against rotavirus | Cincinnati 2011 | Level I |  |  |  | ✔ | ✔ | |  |  |  |  |  | |  |  | ✔ |  |  | No Australian primary source  Low impact on management of acute illness | |
| AGE | Children presenting with gastroenteritis and the following clinical signs or symptoms - reduced urine output, thirst and no physical signs, are diagnosed as not dehydrated | NSW Health 2010 | consensus |  |  |  |  | ✔ | |  |  |  |  |  | |  |  |  | ✔ |  | Covered in another indicator – merged. | |
| AGE | Children presenting with gastroenteritis and the following clinical signs or symptoms - reduced urine output, thirst, dry mucous membranes, and mild tachycardia are diagnosed with mild dehydration (3% of body weight). | NSW Health 2010 | consensus | ✔ |  |  |  | ✔ | |  |  |  |  |  | |  |  |  | ✔ |  | Covered in another indicator – merged. | |
| AGE | Children presenting with gastroenteritis and mild signs plus the following signs - tachycardia, abnormal respiratory pattern, lethargy, reduced skin turgor, sunken eyes are diagnosed with moderate dehydration (5% of body weight). | NSW Health 2010 | consensus | ✔ |  |  |  | ✔ | |  |  |  |  |  | |  |  |  | ✔ |  | Covered in another indicator – merged. | |
| AGE | Children presenting with gastroenteritis and moderate signs plus the following signs - poor perfusion (skin mottled, cool limbs, slow capillary refill, altered consciousness), shock (thready peripheral pulses with marked tachycardia and other signs of poor perfusion) are diagnosed with severe dehydration (10% of body weight) | NSW Health 2010 | consensus | ✔ |  |  |  | ✔ | |  |  |  |  |  | |  |  |  | ✔ |  | Covered in another indicator – merged. | |
| AGE | Children presenting to the GP with gastroenteritis should be referred to ED/hospital if: - if the diagnosis is in doubt - infants/children have moderate or severe dehydration - there are electrolyte abnormalities - they are at high risk of dehydration on the basis of age (<6mths) with a high frequency of diarrhoea (8 in 24hrs) and vomiting (>4 in 24hrs). They should be observed for 4-6hrs to ensure adequate maintenance of hydration -they are high risk infants/children (ileostomy, short gut, cyanotic heart disease, chronic renal disease, metabolic disorders and malnutrition) - infants/children whose parents and carers are thought to be unable to manage the child’s condition at home - IV fluids are required for more than 24hrs | Cincinnati 2011  Heinz 2008  RCH Melb 2013a  RCH paed handbook 2009 | consensus |  | ✔ |  |  | ✔ | |  |  |  |  |  | |  | ✔ |  |  |  | Too complex to measure. | |
| AGE | Children admitted to hospital with gastroenteritis and severe clinical signs of dehydration should receive: - oxygen until signs of shock are reversed. - IV access (use the intraosseous route if required) - blood tests: EUC, BGL (if possible)  - IV bolus of 20mL/kg 0.9% NaCl or Hartmanns stat. - regular assessments for signs of shock  - a repeat IV fluid bolus if necessary until signs of shock are reversed (organ perfusion restored), if >40ml/kg boluses required involve senior staff and ICU - ORS once initial resus complete (infants =30ml/hr, toddlers =60ml/hr, older children =90ml/hr + 10ml/kg for every loose stool or vomit) - their hydration status assessed. Based on this assessment administer IV fluid replacement over 24 hours: 0.9% NaCl + 2.5% Glucose or 0.45% NaCl + 2.5% Glucose  - frequent assessment of fluid balance and clinical assessment  - continuous monitoring | Churgay 2012  Kelly 2007  NICE 2009  Noone 2012  NSW Health 2010  RCH Melb 2013b  RCH Paed 2009  WCH 2010 | consensus |  | ✔ |  |  | ✔ | |  |  |  |  |  | |  |  |  | ✔ |  | Covered in another indicator – merged. | |
| AGE | Children presenting with gastroenteritis and no/mild clinical signs of dehydration should receive parental advice about planned follow-up and review if there is a failure to improve, deterioration or development of new signs | NSW Health 2010 | consensus |  |  | ✔ |  | ✔ | |  |  |  |  |  | |  | ✔ |  |  |  | Unlikely to be documented. | |

**References:**

Churgay 2012: Churgay CA, Aftab Z. Gastroenteritis in children: Part II. prevention and management. American Family Physician. 2012;85(11):1066-70.

Cincinnati 2011: Acute Gastroenteritis Guideline Team Cincinnati Children's hospital medical center. Evidence based care guideline -Prevention and management of Acute Gastroenteritis (AGE) in children aged 2 months to 18 years Cincinnati, Ohio 2011.

Heinz 2008: Heinz, P. Management of acute gastroenteritis in children. Paediatrics and Child Health. 2008;18(10):453-7.

Kelly 2007: Kelly A, Cheong, E. Paediatric gastroenteritis, Australian Doctors Group, Sydney 2007

NICE 2009: National Institute for Health and Clinical Excellence (NICE). Diarrhoea and vomiting in children Diarrhoea and vomiting caused by gastroenteritis: diagnosis, assessment and management in children younger than 5 years. London 2009.

Noone 2012: Noone M. Management of acute gastroenteritis in children. Paediatrics and Child Health (United Kingdom). 2012;22(10):426-31.

NSW Health 2010: NSW Health. Children and infants with gastroenteritis - acute management, Sydney 2010. Available from:

RCH Melb 2013a: The Royal Children's Hospital Melbourne. Abdominal pain. Melbourne, 2013.

RCH Melb 2013b: The Royal Children's Hospital Melbourne. Gastroenteritis. Melbourne, 2013.

RCH Paed 2009: The Royal Childrens Hospital Melbourne. Paediatric Handbook - Eighth edition. Melbourne, Australia: Wiley-Blackwell; 2009.

WCH 2010: Women's and Children's Health Network. Gastroenteritis. Adelaide, 2010.
