# Supplementary material for: Appropriate management of acute gastroenteritis in Australian children: A population-based study
Source: PLoS One. 2019 Nov 7;14(11):e0224681. doi: 10.1371/journal.pone.0224681 (PMC6837505; doi:10.1371/journal.pone.0224681)
Supplement: S3 Appendix — (DOCX) [file pone.0224681.s003.docx]

**S3 Appendix. Conversion of AGE candidate indicators to medical record indicator questions**

The clinical indicators and items are presented by condition, with their source, the number of reviewers (external wiki review), mean score (SD) for appropriateness, level of evidence, and whether they were measured for under- or over-use.

U / O underuse / overuse

HCP healthcare practice

ED Emergency Department

Hosp Hospital (inpatients)

GP General Practice

Spec Specialist (e.g. general paediatrician)

QI Quality indicator

|  | **Candidate indicator** | **Item No** | **Final indicator/QIs** | **No. of reviewers*** | **Source** | **Level of evidence** | **U / O** | **Classification** | **HCP facilities** |
| --- | --- | --- | --- | --- | --- | --- | --- | --- | --- |
| 1 | Children presenting with gastroenteritis have the following history recorded:  - fluid intake AND  - urine output AND  - frequency of vomiting and diarrhoea AND  - duration of illness. | AGE01 | Children who presented with gastroenteritis had their fluid intake recorded. | 3 | NSW Kids and Families. 2010  Guarino A, et al. 2008  Cincinnati Children's HMC. 2011 | Consensus-based recommendation | U | Diagnosis | ED  GP  Hosp |
|  |  | AGE02 | Children who presented with gastroenteritis had their urine output recorded. | 3 | NSW Kids and Families. 2010.  Guarino A, et al. 2008  Cincinnati Children's HMC. 2011 | Consensus-based recommendation | U | Diagnosis | ED  GP  Hosp |
|  |  | AGE03 | Children who presented with gastroenteritis had the frequency of their vomiting and diarrhoea recorded. | 3 | NSW Kids and Families. 2010.  Guarino A, et al. 2008  Cincinnati Children's HMC. 2011 | Consensus-based recommendation | U | Diagnosis | ED  GP  Hosp |
|  |  | AGE04 | Children who presented with gastroenteritis had the duration of their illness recorded. | 3 | NSW Kids and Families. 2010  Guarino A, et al. 2008  Cincinnati Children's HMC. 2011 | Consensus-based recommendation | U | Diagnosis | ED  GP  Hosp |
| 2 | Children presenting with gastroenteritis have their weight recorded. | AGE05 | Children who presented with gastroenteritis had their weight recorded. | 3 | NSW Kids and Families. 2010  Guarino A, et al. 2008  Cincinnati Children's HMC. 2011 | Consensus-based recommendation | U | Diagnosis | ED  GP  Hosp |
| 3 | Children presenting with gastroenteritis have the following assessed:  - lethargy AND  - mucous membranes AND  - depressed fontanelle in babies (age 0-12 months) AND  - Observations - Temp, Heart Rate, Resp, BP. | AGE06 | Children who presented with gastroenteritis were assessed for lethargy. | 3 | NSW Kids and Families. 2010  Guarino A, et al. 2008  Cincinnati Children's HMC. 2011 | Consensus-based recommendation | U | Diagnosis | ED  GP  Hosp |
|  |  | AGE07 | Children who presented with gastroenteritis had their mucous membranes assessed. | 3 | NSW Kids and Families. 2010  Guarino A, et al.2008  Cincinnati Children's HMC. 2011 | Consensus-based recommendation | U | Diagnosis | ED  GP  Hosp |
|  |  | AGE08 | Babies (aged <12 months) who presented with gastroenteritis had their fontanelle assessed. | 3 | NSW Kids and Families. 2010  Guarino A, et al.2008  Cincinnati Children's HMC. 2011 | Consensus-based recommendation | U | Diagnosis | ED  GP  Hosp |
|  |  | AGE09 | Children who presented with gastroenteritis had their observations (Temp, Heart Rate, Resp, BP) assessed. | 3 | NSW Kids and Families. 2010  Guarino A, et al.2008  Cincinnati Children's HMC. 2011 | Consensus-based recommendation | U | Diagnosis | ED  GP  Hosp |
| 4 | Children presenting with gastroenteritis are assessed to determine the degree of dehydration. | AGE10 | Children who presented with gastroenteritis had their degree of dehydration assessed. | 3 | NSW Kids and Families. 2010  Guarino A, et al.2008  Cincinnati Children's HMC. 2011 | Consensus-based recommendation | U | Diagnosis | ED  GP  Hosp |
| 5 | Children with gastroenteritis presenting to the ED with any of the following receive electrolytes and venous blood gas:  - requiring intravenous therapy (IVT) OR  - severe dehydration OR  - altered conscious state / convulsions OR  - pre-existing medical conditions that predispose to electrolyte abnormalities e.g. cystic fibrosis, renal impairment, diabetes. | AGE11 | Children who presented to the ED with gastroenteritis and required intravenous therapy (IVT), received electrolytes. | 3 | Cincinnati Children's HMC. 2011  Heinz P. 2008 | Consensus-based recommendation | U | Treatment | ED |
|  |  | AGE12 | Children who presented to the ED with gastroenteritis and required intravenous therapy (IVT), received a venous blood gas. | 3 | Cincinnati Children's HMC. 2011  Heinz P. 2008 | Consensus-based recommendation | U | Treatment | ED |
|  |  | AGE13 | Children who presented to the ED with gastroenteritis and severe dehydration, received electrolytes. | 3 | Cincinnati Children's HMC. 2011  Heinz P. 2008 | Consensus-based recommendation | U | Treatment | ED |
|  |  | AGE14 | Children who presented to the ED with gastroenteritis and severe dehydration, received a venous blood gas. | 3 | Cincinnati Children's HMC. 2011  Heinz P. 2008 | Consensus-based recommendation | U | Treatment | ED |
|  |  | AGE15 | Children who presented to the ED with gastroenteritis and altered conscious state / convulsions received electrolytes. | 3 | Cincinnati Children's HMC. 2011  Heinz P. 2008 | Consensus-based recommendation | U | Treatment | ED |
|  |  | AGE16 | Children who presented to the ED with gastroenteritis and altered conscious state / convulsions received a venous blood gas. | 3 | Cincinnati Children's HMC. 2011  Heinz P. 2008 | Consensus-based recommendation | U | Treatment | ED |
|  |  | AGE17 | Children who presented to the ED with gastroenteritis and pre-existing medical conditions that predispose to electrolyte abnormalities (e.g. cystic fibrosis, renal impairment, diabetes), received electrolytes. | 3 | Cincinnati Children's HMC. 2011  Heinz P. 2008 | Consensus-based recommendation | U | Treatment | ED |
|  |  | AGE18 | Children who presented to the ED with gastroenteritis and pre-existing medical conditions that predispose to electrolyte abnormalities (e.g. cystic fibrosis, renal impairment, diabetes), received a venous blood gas. | 3 | Cincinnati Children's HMC. 2011  2011.Heinz P. 2008 | Consensus-based recommendation | U | Treatment | ED |
| 6 | Children with gastroenteritis do NOT receive routine blood tests unless there are signs and symptoms of dehydration. | AGE19 | Children with gastroenteritis and NO signs and symptoms of dehydration, did not receive routine blood tests. | 3 | Cincinnati Children's HMC. 2011 | Consensus-based recommendation | O | Treatment | ED  GP  Hosp |
| 7 | Children with gastroenteritis without signs of infection are NOT prescribed:  - anti-diarrhoeals (such as loperimide, kaolin), OR  - antiemetics other than single dose ondansetron (not maxalon, stemetil, multi-dose ondansetron) OR  - antibiotics. | AGE20 | Children with gastroenteritis and no signs of infection were not prescribed anti-diarrhoeals (such as loperimide, kaolin). | 3 | Cincinnati Children's HMC. 2011  The Royal Children's Hospital Melbourne. 2013  Farthing, et al.2008 | Consensus-based recommendation | O | Treatment | ED  GP  Hosp |
|  |  | AGE21 | Children with gastroenteritis and no signs of infection were not prescribed maxalon, stemetil, multi-dose ondansetron. | 2 | Cincinnati Children's HMC. 2011  The Royal Children's Hospital Melbourne. 2013  Farthing, et al.2008 | Consensus-based recommendation | O | Treatment | ED  GP  Hosp |
|  |  | AGE22 | Children with gastroenteritis and no signs of infection were not prescribed antibiotics. | 3 | Cincinnati Children's HMC. 2011  The Royal Children's Hospital Melbourne. 2013  Farthing, et al.2008 | Consensus-based recommendation | O | Treatment | ED  GP  Hosp |
| 8 | Children presenting with gastroenteritis who are severely dehydrated receive IV fluid rehydration including a 20ml/kg bolus. | AGE23 | Children who presented with gastroenteritis and were severely dehydrated, received IV fluid rehydration including a 20ml/kg bolus. | 3 | Cincinnati Children's HMC. 2011 | Consensus-based recommendation | U | Treatment | ED Hosp |
| 9 | Children presenting with gastroenteritis who have no or mild signs of dehydration and are able to tolerate oral fluids are:  - discharged from hospital AND  - advised to re-present if symptoms are unchanged or worsen, AND  - advised to continue with usual diet, AND/OR  - provided with information on age-appropriate oral fluid replacement (small fluids often; breastfeeding / formula, oral rehydration solution or dilute clear fluids). | AGE24 | Children who presented with gastroenteritis, had no or mild signs of dehydration, and were able to tolerate oral fluids were discharged from hospital. | 3 | NSW Kids and Families. 2010  Sydney Children's Hospital. 2004  National Institute for Health and Care Excellence (NICE). 2009  Churgay CA, Aftab Z. 2012  Women's and Children's Health Network. 2010  Kelly A, Cheong E. 2007  Cincinnati Children's HMC. 2011 | Consensus-based recommendation | U | Ongoing management | ED Hosp |
|  |  | AGE25 | Children who presented with gastroenteritis, had no or mild signs of dehydration, and were able to tolerate oral fluids were advised to re-present if symptoms are unchanged or worsen. | 3 | NSW Kids and Families. 2010  Sydney Children's Hospital. 2004  National Institute for Health and Care Excellence (NICE). 2009  Churgay CA, Aftab Z. 2012  Women's and Children's Health Network. 2010  Kelly A, Cheong E. 2007  Cincinnati Children's HMC. 2011 | Consensus-based recommendation | U | Ongoing management | ED  GP  Hosp |
|  |  | AGE26 | Children who presented with gastroenteritis, had no or mild signs of dehydration, and were able to tolerate oral fluids were advised to continue with usual diet. | 3 | NSW Kids and Families. 2010  Sydney Children's Hospital. 2004  National Institute for Health and Care Excellence (NICE). 2009  Churgay CA, Aftab Z. 2012  Women's and Children's Health Network. 2010  Kelly A, Cheong E. 2007  Cincinnati Children's HMC. 2011 | Consensus-based recommendation | U | Ongoing management | ED  GP  Hosp |
|  |  | AGE27 | Children who presented with gastroenteritis, had no or mild signs of dehydration, and were able to tolerate oral fluids were provided with information on age-appropriate oral fluid replacement (small fluids often; breastfeeding / formula, oral rehydration solution or dilute clear fluids). | 3 | NSW Kids and Families. 2010  Sydney Children's Hospital. 2004  National Institute for Health and Care Excellence (NICE). 2009  Churgay CA, Aftab Z. 2012  Women's and Children's Health Network. 2010  Kelly A, Cheong E. 2007  Cincinnati Children's HMC. 2011 | Consensus-based recommendation | U | Ongoing management | ED  GP  Hosp |
| 10 | Children presenting to the GP with gastroenteritis who have any of the following are referred to hospital or the ED:  - moderate or severe dehydration. | AGE28 | Children who presented to the GP with gastroenteritis and moderate or severe dehydration were referred to hospital or the ED. | 3 | NSW Kids and Families. 2010 | Consensus-based recommendation | U | Ongoing management | GP |
| 11 | Children with gastroenteritis who are moderately to severely dehydrated AND have received rehydration are clinically reassessed within 6hrs for the following:  - weight AND  - clinical signs of dehydration AND  - urine output AND  - ongoing diarrhoea / vomiting AND  - signs of fluid overload (puffy face and extremities). | AGE29 | Children who presented with gastroenteritis, were moderately to severely dehydrated AND received rehydration, had their weight reassessed within 6 hours. | 3 | The Royal Children's Hospital Melbourne. 2013 | Consensus-based recommendation | U | Ongoing management | ED Hosp |
|  |  | AGE30 | Children who presented with gastroenteritis, were moderately to severely dehydrated AND received rehydration, were reassessed for clinical signs of dehydration within 6 hours. | 3 | The Royal Children's Hospital Melbourne. 2013 | Consensus-based recommendation | U | Ongoing management | ED Hosp |
|  |  | AGE31 | Children who presented with gastroenteritis, were moderately to severely dehydrated AND received rehydration, had their urine output reassessed within 6 hours. | 3 | The Royal Children's Hospital Melbourne. 2013 | Consensus-based recommendation | U | Ongoing management | ED Hosp |
|  |  | AGE32 | Children who presented with gastroenteritis, were moderately to severely dehydrated AND received rehydration, were reassessed for ongoing diarrhoea / vomiting within 6 hours. | 3 | The Royal Children's Hospital Melbourne. 2013 | Consensus-based recommendation | U | Ongoing management | ED Hosp |
|  |  | AGE33 | Children who presented with gastroenteritis, were moderately to severely dehydrated AND received rehydration, were reassessed for signs of fluid overload (puffy face and extremities) within 6 hours. | 3 | The Royal Children's Hospital Melbourne. 2013 | Consensus-based recommendation | U | Ongoing management | ED Hosp |
| 12 | Children with gastroenteritis and the following are discharged (even if there is some vomiting):  - sufficient rehydration achieved as indicated by weight gain and/or clinical status (child is rehydrated or only mildly dehydrated) AND"  - gastrointestinal loss is not profuse (oral intake equals or exceeds losses) AND | AGE34 | Children with gastroenteritis who were sufficiently rehydrated as indicated by weight gain and/or clinical status (child is rehydrated or only mildly dehydrated) were discharged. | 3 | NSW Kids and Families. 2010  Cincinnati Children's HMC. 2011 | Consensus-based recommendation | U | Ongoing management | ED Hosp |
|  |  | AGE35 | Children with gastroenteritis who had gastrointestinal loss that was not profuse (oral intake equals or exceeds losses), were discharged. | 3 | NSW Kids and Families. 2010  Cincinnati Children's HMC. 2011 | Consensus-based recommendation | U | Ongoing management | ED Hosp |

**References:**

Churgay CA, Aftab Z. Gastroenteritis in children: Part II. prevention and management. American Family Physician. 2012;85(11):1066-70.

Cincinnati Children's HMC, Acute Gastroenteritis Guideline Team. Evidence based care guideline -Prevention and management of Acute Gastroenteritis (AGE) in children aged 2 months to 18 years. Cincinnati, Ohio 2011.

Farthing M, Lindberg G, Dite P, Khalif I, Salazar-Lindo E, Ramakrishna BS, et al. World Gastroenterology Organisation practice guideline: Acute diarrhea WGO Practice Guidelines. 2008:1-28.

Guarino A, Albano F, Ashkenazi S, Gendrel D, Hoekstra JH, Shamir R, et al. European Society for Paediatric Gastroenterology, Hepatology, and Nutrition/European Society for Paediatric Infectious Diseases evidence-based guidelines for the management of acute gastroenteritis in children in Europe: executive summary. Journal of Pediatric Gastroenterology and Nutrition. 2008;46 (5):619-21.

Heinz P. Management of acute gastroenteritis in children. Paediatrics and Child Health. 2008;18(10):453-7.

Kelly A, Cheong, E. Paediatric gastroenteritis, Australian Doctor Group, Sydney 2007.

National Institute for Health and Clinical Excellence (NICE). Diarrhoea and vomiting in children Diarrhoea and vomiting caused by gastroenteritis: diagnosis, assessment and management in children younger than 5 years, London 2009.

NSW Kids and Families. Children and infants with gastroenteritis - acute management 2010.

Sydney Children's Hospital. Gastroenteritis Clinical Guideline, Sydney 2004.

The Royal Children's Hospital Melbourne. Gastroenteritis Clinical Guideline, Melbourne 2013.

Women's and Children's Health Network. Gastroenteritis, Adelaide 2010.
