# Supplementary material for: Appropriate management of acute gastroenteritis in Australian children: A population-based study
Source: PLoS One. 2019 Nov 7;14(11):e0224681. doi: 10.1371/journal.pone.0224681 (PMC6837505; doi:10.1371/journal.pone.0224681)
Supplement: S4 Appendix — (DOCX) [file pone.0224681.s004.docx]

**S5 Appendix. Key information from the CareTrack Kids Surveyors’ Manual relevant to assessment of AGE indicators**

# General rules

1. Before commencing each medical record review, please check:
   1. Patient identification – is this the correct patient? (patient ID, gender and date of birth)
   2. Condition listed in the medical record – does this match the condition the patient was selected for?
   3. Date of medical record visit – is this during the study period, i.e. 2012 -2013?
   4. The point of care / contact for each condition and healthcare practitioner (HCP) – is this the first encounter for this condition and presentation to this HCP? Does this visit correlate with diagnosis of the condition, or is this a follow-up visit?
2. Screen the entire medical record for additional sources of information, relevant for the time period:
   1. History – e.g. past medical, family/social
   2. Allergies – e.g. drug allergies, reactions to topical agents
   3. Test results - e.g. ABGs, blood tests, radiology, letters and other correspondence
3. Read indicator carefully. Pay particular attention to:
   1. Inclusion criteria – shaded in red
   2. Compliance action(s) – shaded in yellow
   3. Any definitions
   4. Get a sense of the “spirit” or intent of the indicator – e.g. exactly what is being asked? Is it assessing under-use or over-servicing (NOTE: most indicators assess under-use; over-servicing type indicators are marked with an *)?
4. If it’s not written down, it did not happen. Do not make assumptions.
5. In the following circumstances, documentation by exclusion is sufficient for compliance (e.g. HCPs do not need to re-record this information at each visit):
   1. History – e.g. past medical, family/social
   2. Allergies – e.g. drug allergies, reactions to topical agents
   3. Test results - e.g. ABGs, blood tests, radiology, letters and other correspondence
6. All indicators that relate to care provided “at diagnosis” are assessed at the first visit/encounter for this condition, at each HCP - e.g. first visit to the GP AND first visit to the specialist(s)
7. All indicators that relate to “first-line treatment” can only ever occur ONCE for each patient – e.g. if reviewing a specialists’ record, and patient has already been provided with treatment from a different HCP for this same condition, this will be marked as NA
8. All indicators with reference to a timeframe, please consider this a minimum requirement unless otherwise stated – e.g. “are reviewed every 6 months”, means that we are assessing compliance for at least one review occurring within the 6 months.
9. In the absence of definitions, use your clinical judgement to determine what is appropriate and practical to occur, and note this information in the “Comments” field (e.g. Medical record states there is no past medical history (i.e. nil) – which may not be updated at each visit, however a recent new illness should be documented at this visit)
10. If there are multiple visits in a short period of time – it will be your responsibility to use your clinical judgment to determine if it is reasonable to expect the HCP to complete all care again. In the case of some conditions or indicators it may not be appropriate. For example if a patient visited an ED repeatedly in a small timeframe (i.e. 5, 6 and 7^th^ June 2012) for anxiety - it may be appropriate for indicators such as “Children who presented with suspected anxiety had their family circumstances assessed” for the HCP to have written in the clinical notes “as per visit on 5/6”.
11. The presenting condition is audited for each visit. However if the diagnosis changes (i.e presented with Abdominal Pain and part-way through the visit is diagnosed with Acute Gastroenteritis) then from that point of time onwards, all questions for abdominal pain become NA. If the new diagnosis is a CTK condition and care is continuing at the same HCP, the surveyor will then edit visit dates and add a new visit in from that point for Acute Gastroenteritis.
12. Ideally, compliance for “monitored” will require both an initial (baseline) assessment and review over time (which, depending on the condition indicators, may be periodical or within a stipulated timeframe). However, there may be some instances where one of the other of these two criteria are not possible or appropriate, or may not be specifically recorded as such (e.g. review of treatment goals, or management). In these instances, you will need to use your clinical judgement to score the indicators. Importantly, populating the “Comments” field with relevant information about what has been documented and your decision-making process, will assist us with ongoing quality control and final data analysis.

# General definitions

## Blood Tests

‘Routine bloods’ may be documented in a medical record, however surveyors cannot assume that specific tests are included under this umbrella-type term. Therefore, in order to mark compliance for the ordering of specific tests (i.e. BGL for Acute Bronchiolitis, TSH for Diabetes, etc.), surveyors must screen the record to locate the results of these specific blood test results, appropriate to the relevant presentation date.

## Discharge

All indicators and questions mentioning “discharged” refer only to the hospital setting and ED.

## Factsheet

This includes any written information provided by the HCP – it does not have to be an official factsheet.

## Greater than >

Greater than is represented in the indicators by the following symbol: **>**. For example >5 years means children aged greater than 5 years.

## Less than <

Less than is represented in the indicators by the following symbol: **<**. For example <2 years means children aged less than 2 years.

## Prescribed

A drug will count as prescribed if it is newly prescribed, currently or continuing to be prescribed, or undergoing a dosage revision.

## Presented with

Some indicator inclusion criteria state “Children who presented with XYZ were…” Presenting with is defined as any visit where the child had the stated inclusion criteria. This includes a first presentation or review presentations for a condition.

## Severity

The severity level of a condition may not be documented in the medical record under an explicit level. In this case use the tables provided in this document to determine the severity level (NOTE: most are condition-specific).

## Side effects

For indicators relating to “monitoring for side effects” this commences at prescribing of medications and documentation indicating that patients are made aware of what to look for in terms of side effects should be located in the clinical record for that visit.

## Visit

In this study, “visit” includes those attendances at a healthcare facility where some form of interaction between the health care provider and the patient occurred. This does not include entries where a script was collected, a letter to or from a specialist was sent or received, or results were entered. Visits also include presentations to an emergency department, without admission to hospital.

# Australasian Harmonised Reference Intervals for Paediatrics

| **Analyte** | **Age** | | **Reference Interval** | |
| --- | --- | --- | --- | --- |
| Sodium | 0w to <1w | | 132 – 147 mmol/L | |
|  | 1w to <18y | | 133 – 144 mmol/L | |
| Potassium (serum) | 0w to <1w | | 3.8 – 6.5 mmol/L | |
|  | 1w to <26w | | 4.2 – 6.7 mmol/L | |
|  | 26w to <2y | | 3.9 – 5.6 mmol/L | |
|  | 2y to <18y | | 3.6 – 5.3 mmol/L | |
| Potassium (plasma) | 0w to <1w | | 3.5 – 6.2 mmol/L | |
|  | 1w to <26w | | 3.8 – 6.4 mmol/L | |
|  | 26w to <2y | | 3.5 – 5.4 mmol/L | |
|  | 2y to <18y | | 3.3 – 4.9 mmol/L | |
| Chloride | 0w to <1w | | 98 – 115 mmol/L | |
|  | 1w to <18y | | 97 – 110 mmol/L | |
| Bicarbonate | 0w to <1w | | 15 – 28 mmol/L | |
|  | 1w to <2y | | 16 – 29 mmol/L | |
|  | 2y to <10y | | 17 – 30 mmol/L | |
|  | 10y to <18y | | 20 – 32 mmol/L | |
| Calcium | 0w to <1w | | 1.85 – 2.80 mmol/L | |
|  | 1w to <26w | | 2.20 – 2.80 mmol/L | |
|  | 26w to <2y | | 2.20 – 2.70 mmol/L | |
|  | 2y to <18y | | 2.20 – 2.65 mmol/L | |
| Magnesium | 0w to <1w | | 0.60 – 1.00 mmol/L | |
|  | 1w to <18y | | 0.65 – 1.10 mmol/L | |
| Phosphate | 0w to <1w | | 1.25 – 2.85 mmol/L | |
|  | 1w to <4w | | 1.50 – 2.75 mmol/L | |
|  | 4w to <26w | | 1.45 – 2.50 mmol/L | |
|  | 26w to <1y | | 1.30 – 2.30 mmol/L | |
|  | 1y to < 4y | | 1.10 – 2.20 mmol/L | |
|  | 4y to <15y | | 0.90 – 2.00 mmol/L | |
|  | 15y to <18y | | 0.80 – 1.85 mmol/L | |
| Creatinine*** | 0w to <1w | | 22 – 93 µmol/L | |
|  | 1w to <4w | | 17 – 50 µmol/L | |
|  | 4w to <2y | | 11 – 36 µmol/L | |
|  | 2y to <6y | | 20 – 44 µmol/L | |
|  | 6y to <12y | | 27 – 58 µmol/L | |
|  | Male | | Female | |
|  | 12y to <15y | 35 – 83 µmol/L | 12y to <15y | 35 – 74 µmol/L |
|  | 15y to <19y | 50 – 100 µmol/L | 15y to <19y | 38 – 82 µmol/L |
| Alkaline phosphate | 0w to <1w | | 80 – 380 U/L | |
|  | 1w to <4w | | 120 – 550 U/L | |
|  | 4w to <26w | | 120 – 650 U/L | |
|  | 26w to <2y | | 120 – 450 U/L | |
|  | 2y to <6y | | 120 – 370 U/L | |
|  | 6y to <10y | | 120 – 440 U/L | |
|  | Male | | Female | |
|  | 10y to <14y | 130 – 530 U/L | 10y to <13y | 100 – 460 U/L |
|  | 14y to <15y | 105 – 480 U/L | 13y to <14y | 70 – 330 U/L |
|  | 15y to <17y | 80 – 380 U/L | 14y to <15y | 50 – 280 U/L |
|  | 17y to <19y | 50 – 220 U/L | 15y to <16y | – 170 U/L |

# Condition specific definitions: Acute gastroenteritis (AGE)

**Degree of dehydration:** Description of dehydration (% of body weight)

- *Mild (3%)*
  - Reduced urine output
  - Thirst
  - Dry mucous membranes
  - Mild tachycardia
- M*oderate (5%)*
  - Dry mucous membranes
  - Tachycardia
  - Abnormal respiratory pattern
  - Lethargy
  - Reduced skin turgor
  - Sunken eyes
- *Severe (10%)*
  - Above signs
  - Poor perfusion: mottled, cool limbs, slow capillary refill, altered consciousness
  - Shock: thready peripheral pulses with marked tachycardia and other signs of poor perfusion stated above

**Observations:** Temperature AND Heart rate AND Respiratory Rate AND BP

**Pre-existing medical conditions that predispose to electrolyte abnormalities:** e.g. cystic fibrosis, renal impairment, diabetes.

**Signs of infections:** fever/temp (over 38°c) and one of more of the following

•         Looks unwell / toxic

•         WCC <500 or >15000

•         Other abnormal test results (CXR / urine / blood culture)

# CTK database questions relevant to AGE

Text highlighted in red is the inclusion criteria. Text highlighted in yellow is the compliance point/s.

| **Condition** | **Question No** | **Question** |
| --- | --- | --- |
| AGE | AGE01 | Children who presented with gastroenteritis had their fluid intake recorded. |
| AGE | AGE02 | Children who presented with gastroenteritis had their urine output recorded. |
| AGE | AGE03 | Children who presented with gastroenteritis had the frequency of their vomiting and diarrhoea recorded. |
| AGE | AGE04 | Children who presented with gastroenteritis had the duration of their illness recorded. |
| AGE | AGE05 | Children who presented with gastroenteritis had their weight recorded. |
| AGE | AGE06 | Children who presented with AGE were assessed for lethargy. |
| AGE | AGE07 | Children who presented with gastroenteritis had their mucous membranes assessed. |
| AGE | AGE08 | Babies (aged 0-12 months) who presented with gastroenteritis had their fontanelle assessed. |
| AGE | AGE09 | Children who presented with gastroenteritis had their observations (Temp, Heart Rate, Resp, BP) assessed. |
| AGE | AGE10 | Children who presented with gastroenteritis had their degree of dehydration assessed. |
| AGE | AGE11 | Children who presented to the ED with gastroenteritis and required intravenous therapy (IVT), received electrolytes. |
| AGE | AGE12 | Children who presented to the ED with gastroenteritis and required intravenous therapy (IVT), received a venous blood gas. |
| AGE | AGE13 | Children who presented to the ED with gastroenteritis and severe dehydration, received electrolytes. |
| AGE | AGE14 | Children who presented to the ED with gastroenteritis and severe dehydration, received a venous blood gas. |
| AGE | AGE15 | Children who presented to the ED with gastroenteritis and altered conscious state / convulsions received electrolytes. |
| AGE | AGE16 | Children who presented to the ED with gastroenteritis and altered conscious state / convulsions received a venous blood gas. |
| AGE | AGE17 | Children who presented to the ED with gastroenteritis and pre-existing medical conditions that predispose to electrolyte abnormalities (e.g. cystic fibrosis, renal impairment, diabetes), received electrolytes. |
| AGE | AGE18 | Children who presented to the ED with gastroenteritis and pre-existing medical conditions that predispose to electrolyte abnormalities (e.g. cystic fibrosis, renal impairment, diabetes), received a venous blood gas. |
| AGE | AGE19 | Children with gastroenteritis and NO signs and symptoms of dehydration, received routine blood tests. |
| AGE | AGE20 | Children with gastroenteritis and no signs of infection were prescribed anti-diarrhoeals (such as loperimide, kaolin). |
| AGE | AGE21 | Children with gastroenteritis and no signs of infection were prescribed antiemetics (but NOT maxalon, stemetil, multi-dose ondansetron). |
| AGE | AGE22 | Children with gastroenteritis and no signs of infection were prescribed antibiotics. |
| AGE | AGE23 | Children who presented with gastroenteritis and were severely dehydrated, received IV fluid rehydration including a 20ml/kg bolus. |
| **Classification - Ongoing Management** | | |
| AGE | AGE24 | Children who presented with gastroenteritis, had no or mild signs of dehydration, and were able to tolerate oral fluids were discharged from hospital. |
| AGE | AGE25 | Children who presented with gastroenteritis, had no or mild signs of dehydration, and were able to tolerate oral fluids were advised to re-present if symptoms are unchanged or worsen. |
| AGE | AGE26 | Children who presented with gastroenteritis, had no or mild signs of dehydration, and were able to tolerate oral fluids were advised to continue with usual diet. |
| AGE | AGE27 | Children who presented with gastroenteritis, had no or mild signs of dehydration, were provided with information on age-appropriate oral fluid replacement (small fluids often; breastfeeding / formula, oral rehydration solution or dilute clear fluids). |
| AGE | AGE28 | Children who presented to the GP with gastroenteritis and moderate or severe dehydration were referred to hospital or the ED. |
| AGE | AGE29 | Children who presented with gastroenteritis, were moderately to severely dehydrated AND received rehydration, had their weight reassessed within 6 hours. |
| AGE | AGE30 | Children who presented with gastroenteritis, were moderately to severely dehydrated AND received rehydration, were reassessed for clinical signs of dehydration within 6 hours. |
| AGE | AGE31 | Children who presented with gastroenteritis, were moderately to severely dehydrated AND received rehydration, had their urine output reassessed within 6 hours. |
| AGE | AGE32 | Children who presented with gastroenteritis, were moderately to severely dehydrated AND received rehydration, were reassessed for ongoing diarrhoea / vomiting within 6 hours. |
| AGE | AGE33 | Children who presented with gastroenteritis, were moderately to severely dehydrated AND received rehydration, were reassessed for signs of fluid overload (puffy face and extremities) within 6 hours. |
| AGE | AGE34 | Children with gastroenteritis who were sufficiently rehydrated as indicated by weight gain and/or clinical status (child is rehydrated or only mildly dehydrated) were discharged. |
| AGE | AGE35 | Children with gastroenteritis who had gastrointestinal loss that was not profuse (oral intake equals or exceeds losses), were discharged. |
