# Supplementary material for: Appropriate management of acute gastroenteritis in Australian children: A population-based study
Source: PLoS One. 2019 Nov 7;14(11):e0224681. doi: 10.1371/journal.pone.0224681 (PMC6837505; doi:10.1371/journal.pone.0224681)
Supplement: S1 Table — (DOCX) [file pone.0224681.s005.docx]

**S4 Table: Characteristics of final clinical indicators for Acute Gastroenteritis**

|  | | | **No. of Sites** | | |  | | |
| --- | --- | --- | --- | --- | --- | --- | --- | --- |
| **Indicator ID** | **Indicator Description** | **Age Inclusion Criteria** | **GP** | **ED** | **INPT** | **Strength of Recommendation or Level of Evidence^#^** | **Phase of Care** | **Quality Type*** |
| AGE01 | Children who presented with gastroenteritis had their fluid intake recorded.^[1-3]^ | 0 - 15 years | 75 | 34 | 26 | Consensus-based recommendation | Diagnosis | Underuse |
| AGE02 | Children who presented with gastroenteritis had their urine output recorded. ^[1-3]^ | 0 - 15 years | 75 | 34 | 26 | Consensus-based recommendation | Diagnosis | Underuse |
| AGE03 | Children who presented with gastroenteritis had the frequency of their vomiting and diarrhoea recorded.^[1-3]^ | 0 - 15 years | 75 | 34 | 26 | Consensus-based recommendation | Diagnosis | Underuse |
| AGE04 | Children who presented with gastroenteritis had the duration of their illness recorded.^[1-3]^ | 0 - 15 years | 75 | 34 | 26 | Consensus-based recommendation | Diagnosis | Underuse |
| AGE05 | Children who presented with gastroenteritis had their weight recorded.^[1-3]^ | 0 - 15 years | 75 | 34 | 26 | Consensus-based recommendation | Diagnosis | Underuse |
| AGE06 | Children who presented with gastroenteritis were assessed for lethargy.^[1-3]^ | 0 - 15 years | 75 | 34 | 26 | Consensus-based recommendation | Diagnosis | Underuse |
| AGE07 | Children who presented with gastroenteritis had their mucous membranes assessed.^[1-3]^ | 0 - 15 years | 75 | 34 | 26 | Consensus-based recommendation | Diagnosis | Underuse |
| AGE08 | Babies (aged < 12 months) who presented with gastroenteritis had their fontanelle assessed.^[1-3]^ | 0 - 11 months | 24 | 31 | 18 | Consensus-based recommendation | Diagnosis | Underuse |
| AGE09 | Children who presented with gastroenteritis had their observations (Temp, HR, RR, BP) assessed.^[1-3]^ | 0 - 15 years | 75 | 34 | 26 | Consensus-based recommendation | Diagnosis | Underuse |
| AGE10 | Children who presented with gastroenteritis had their degree of dehydration assessed.^[1-3]^ | 0 - 15 years | 75 | 34 | 26 | Consensus-based recommendation | Diagnosis | Underuse |
| AGE11 | Children who presented to the ED with gastroenteritis and required intravenous therapy, received electrolytes.^[3,4]^ | 0 - 15 years | NA | 31 | NA | Consensus-based recommendation | Treatment | Underuse |
| AGE12 | Children who presented to the ED with gastroenteritis and required intravenous therapy, received a venous blood gas.^[3,4]^ | 0 - 15 years | NA | 31 | NA | Consensus-based recommendation | Treatment | Underuse |
| AGE13 | Children who presented to the ED with gastroenteritis and severe dehydration, received electrolytes.^[3,4]^ | 0 - 15 years | NA | 12 | NA | Consensus-based recommendation | Treatment | Underuse |
| AGE14 | Children who presented to the ED with gastroenteritis and severe dehydration, received a venous blood gas.^[3,4]^ | 0 - 15 years | NA | 12 | NA | Consensus-based recommendation | Treatment | Underuse |
| AGE15 | Children who presented to the ED with gastroenteritis and altered conscious state/convulsions received electrolytes.^[3,4]^ | 0 - 15 years | NA | 7 | NA | Consensus-based recommendation | Treatment | Underuse |
| AGE16 | Children who presented to the ED with gastroenteritis and altered conscious state/convulsions received a venous blood gas.^[3,4]^ | 0 - 15 years | NA | 6 | NA | Consensus-based recommendation | Treatment | Underuse |
| AGE17 | Children who presented to the ED with gastroenteritis and pre-existing medical conditions that predispose to electrolyte abnormalities (e.g. cystic fibrosis, renal impairment, diabetes), received electrolytes.^[3,4]^ | 0 - 15 years | NA | 15 | NA | Consensus-based recommendation | Treatment | Underuse |
| AGE18 | Children who presented to the ED with gastroenteritis and pre-existing medical conditions that predispose to electrolyte abnormalities (e.g. cystic fibrosis, renal impairment, diabetes), received a venous blood gas.^[3,4]^ | 0 - 15 years | NA | 15 | NA | Consensus-based recommendation | Treatment | Underuse |
| AGE19 | Children with gastroenteritis and NO signs and symptoms of dehydration, did not receive routine blood tests.^[3]^ | 0 - 15 years | 72 | 33 | 19 | Consensus-based recommendation | Treatment | Overuse |
| AGE20 | Children with gastroenteritis and no signs of infection were not prescribed anti-diarrhoeals (such as loperimide, kaolin).^[3,5,6]^ | 0 - 15 years | 74 | 34 | 26 | Consensus-based recommendation | Treatment | Overuse |
| AGE21 | Children with gastroenteritis and no signs of infection were not prescribed maxalon, stemetil, multi-dose ondansetron.^[3,5,6]^ | 0 - 15 years | 74 | 34 | 26 | Consensus-based recommendation | Treatment | Overuse |
| AGE22 | Children with gastroenteritis and no signs of infection were not prescribed antibiotics.^[3,5,6]^ | 0 - 15 years | 74 | 34 | 26 | Consensus-based recommendation | Treatment | Overuse |
| AGE23 | Children who presented with gastroenteritis and were severely dehydrated, received IV fluid rehydration including a 20 ml/kg bolus.^[3]^ | 0 - 15 years | NA | 14 | 11 | Consensus-based recommendation | Treatment | Underuse |
| AGE24 | Children who presented with gastroenteritis, had no or mild signs of dehydration, and were able to tolerate oral fluids were discharged from hospital.^[1,3,7-11]^ | 0 - 15 years | NA | 33 | 24 | Consensus-based recommendation | Ongoing management | Underuse |
| AGE25 | Children who presented with gastroenteritis, had no or mild signs of dehydration, and were able to tolerate oral fluids were advised to re-present if symptoms are unchanged or worsen.^[1,3,7-11]^ | 0 - 15 years | 73 | 33 | 24 | Consensus-based recommendation | Ongoing management | Underuse |
| AGE26 | Children who presented with gastroenteritis, had no or mild signs of dehydration, and were able to tolerate oral fluids were advised to continue with usual diet.^[1,3,7-11]^ | 0 - 15 years | 74 | 33 | 24 | Consensus-based recommendation | Ongoing management | Underuse |
| AGE27 | Children who presented with gastroenteritis, had no or mild signs of dehydration, and were able to tolerate oral fluids were provided with information on age-appropriate oral fluid replacement (small fluids often; breastfeeding/formula, oral rehydration solution or dilute clear fluids).^[1,3,7-11]^ | 0 - 15 years | 74 | 33 | 24 | Consensus-based recommendation | Ongoing management | Underuse |
| AGE28 | Children who presented to the GP with gastroenteritis and moderate or severe dehydration were referred to hospital or the ED.^[1]^ | 0 - 15 years | 12 | NA | NA | Consensus-based recommendation | Ongoing management | Underuse |
| AGE29 | Children who presented with gastroenteritis, were moderately to severely dehydrated AND received rehydration, had their weight reassessed within 6 hours.^[5]^ | 0 - 15 years | NA | 28 | 19 | Consensus-based recommendation | Ongoing management | Underuse |
| AGE30 | Children who presented with gastroenteritis, were moderately to severely dehydrated AND received rehydration, were reassessed for clinical signs of dehydration within 6 hours.^[5]^ | 0 - 15 years | NA | 28 | 19 | Consensus-based recommendation | Ongoing management | Underuse |
| AGE31 | Children who presented with gastroenteritis, were moderately to severely dehydrated AND received rehydration, had their urine output reassessed within 6 hours.^[5]^ | 0 - 15 years | NA | 28 | 19 | Consensus-based recommendation | Ongoing management | Underuse |
| AGE32 | Children who presented with gastroenteritis, were moderately to severely dehydrated AND received rehydration, were reassessed for ongoing diarhhoea/vomiting within 6 hours.^[5]^ | 0 - 15 years | NA | 28 | 19 | Consensus-based recommendation | Ongoing management | Underuse |
| AGE33 | Children who presented with gastroenteritis, were moderately to severely dehydrated AND received rehydration, were reassessed for signs of fluid overload (puffy face and extremities) within 6 hours.^[5]^ | 0 - 15 years | NA | 28 | 19 | Consensus-based recommendation | Ongoing management | Underuse |
| AGE34 | Children with gastroenteritis who were sufficiently rehydrated as indicated by weight gain and/or clinical status (child is rehydrated or only mildly dehydrated) were discharged.^[1,3]^ | 0 - 15 years | NA | 30 | 26 | Consensus-based recommendation | Ongoing management | Underuse |
| AGE35 | Children with gastroenteritis who had gastrointestinal loss that was not profuse (oral intake equals or exceeds losses), were discharged.^[1,3]^ | 0 - 15 years | NA | 32 | 26 | Consensus-based recommendation | Ongoing management | Underuse |

**Legend**: ID=Identifier; GP=General Practice; ED=Emergency Department; INPT=Inpatient; HR=Heart Rate; Temp=Temperature; RR=Respiratory Rate; BP=Blood Pressure.

**Source Clinical Practice Guidelines**:

1. NSW Kids and Families. Children and infants with gastroenteritis - acute management (2010).

2. Guarino, A. et al. European Society for Paediatric Gastroenterology, Hepatology, and Nutrition/European Society for Paediatric Infectious Diseases evidence-based guidelines for the management of acute gastroenteritis in children in Europe: executive summary. Journal of Pediatric Gastroenterology and Nutrition 46 (5), 619-621 (2008).

3. Acute Gastroenteritis Guideline Team Cincinnati Children's hospital medical center. Evidence based care guideline -Prevention and management of Acute Gastroenteritis (AGE) in children aged 2 months to 18 years (2011).

4. Heinz, P. Management of acute gastroenteritis in children. Paediatrics and Child Health 18, 453-457 (2008).

5. The Royal Children's Hospital Melbourne. Gastroenteritis (2013).

6. Farthing, M. et al. World Gastroenterology Organisation practice guideline: Acute diarrhea WGO Practice Guidelines, 1-28 (2008).

7. Sydney Children's Hospital. Gastroenteritis Clinical Guideline (2004).

8. National Institute for Health and Clinical Excellence (NICE). Diarrhoea and vomiting in children. Diarrhoea and vomiting caused by gastroenteritis: diagnosis, assessment and management in children younger than 5 years (2009).

9. Churgay, C. A. & Aftab, Z. Gastroenteritis in children: Part II. prevention and management. American Family Physician 85, 1066-1070 (2012).

10. Women's and Children's Health Network. Gastroenteritis (2010).

11. Kelly, A., Cheong, E. Paediatric gastroenteritis (2007).

# Where Strength of Recommendation (in Grades), or Level of Evidence (Levels), were not specified in the CPG, the term “Consensus-based recommendation” was assigned.

* The type of quality of care assessed was classified as underuse or overuse: underuse refers to actions which are recommended, but not undertaken; overuse refers to actions which are not indicated, or are contraindicated, in the context of the indicator’s inclusion criteria.
